# Development and Psychometric Evaluation of an Instrument to Assess Cross-Cultural Competence of Healthcare Professionals (CCCHP)

**DOI:** 10.1371/journal.pone.0144049

**Published:** 2015-12-07

**Authors:** Gerda Bernhard, Ronald A. Knibbe, Alessa von Wolff, Demet Dingoyan, Holger Schulz, Mike Mösko

**Affiliations:** 1 Department of Medical Psychology, University Medical Center Hamburg-Eppendorf, Hamburg, Germany; 2 Department of Health Promotion, Maastricht University, Maastricht, The Netherlands; Philipps University Marburg, GERMANY

## Abstract

**Background:**

Cultural competence of healthcare professionals (HCPs) is recognized as a strategy to reduce cultural disparities in healthcare. However, standardised, valid and reliable instruments to assess HCPs’ cultural competence are notably lacking. The present study aims to 1) identify the core components of cultural competence from a healthcare perspective, 2) to develop a self-report instrument to assess cultural competence of HCPs and 3) to evaluate the psychometric properties of the new instrument.

**Methods:**

The conceptual model and initial item pool, which were applied to the cross-cultural competence instrument for the healthcare profession (CCCHP), were derived from an expert survey (n = 23), interviews with HCPs (n = 12), and a broad narrative review on assessment instruments and conceptual models of cultural competence. The item pool was reduced systematically, which resulted in a 59-item instrument. A sample of 336 psychologists, in advanced psychotherapeutic training, and 409 medical students participated, in order to evaluate the construct validity and reliability of the CCCHP.

**Results:**

Construct validity was supported by principal component analysis, which led to a 32-item six-component solution with 50% of the total variance explained. The different dimensions of HCPs’ cultural competence are: Cross-Cultural Motivation/Curiosity, Cross-Cultural Attitudes, Cross-Cultural Skills, Cross-Cultural Knowledge/Awareness and Cross-Cultural Emotions/Empathy. For the total instrument, the internal consistency reliability was .87 and the dimension’s Cronbach’s α ranged from .54 to .84. The discriminating power of the CCCHP was indicated by statistically significant mean differences in CCCHP subscale scores between predefined groups.

**Conclusions:**

The 32-item CCCHP exhibits acceptable psychometric properties, particularly content and construct validity to examine HCPs’ cultural competence. The CCCHP with its five dimensions offers a comprehensive assessment of HCPs’ cultural competence, and has the ability to distinguish between groups that are expected to differ in cultural competence. This instrument can foster professional development through systematic self-assessment and thus contributes to improve the quality of patient care.

## Introduction

European countries, including Germany, are becoming increasingly ethnically and culturally diverse, resulting from the rise of international immigration and asylum applications. Inequalities in health, and access to healthcare between migrants and local populations in Europe, have been noted [1]. Many studies have suggested that cultural differences influence communication between patients and providers [2], resulting in inadequate diagnostic testing [3], miscommunications about etiologies, inadequate treatment plans [4], and disregard of patients’ thoughts and ideas [5].

Cultural competence has gained national and international attention as a strategy to reduce cultural disparities in health and healthcare [6]. The utilization of cultural competency training as a means to improve the cultural competence of healthcare providers and address health disparities is well documented [7]. However, an often-cited weakness of cultural competency training is the lack of standardised and validated instruments to assess its impact [8, 9]. The assessment of cultural competence of healthcare professionals involved in direct patient care (HCPs; i.e. physicians, clinicians, psychotherapists, psychologists, midwives and nurses) is important to determine individual strengths and weaknesses, leading to self-awareness [10] and is therefore a necessary, effective and systematic way to plan for and integrate cultural competency in healthcare organisations (e.g. hospitals, primary care services) [11].

Although a vast amount of international literature on cultural competence exists, there is considerable confusion about what constitutes as cultural competence. A variety of academic disciplines, including healthcare and nursing [12, 13], counselling [14], social work [15], and other health professions such as occupational therapy [16] and rehabilitation [17] have laid claim to the construct of cultural competence and produced a number of models. The most widely used conceptualisation of culturally competent practice is based on a three-dimensional model (beliefs and attitudes, knowledge, and skills) presented by Sue et al. [18]. While many academics agree that cultural competence comprises knowledge, skills, and attitudes, the definition and operationalisation of cultural competence differs greatly between studies and instruments [19, 20]. Moreover, consultation with members of the target population (HCPs) and experts for the purpose of conceptualizing the construct of cultural competence is underused in existing instruments. Furthermore, the majority of cultural competence models and instruments have been developed in the United States and reflect the socio-cultural and political context in which they were developed [21, 22]. Thus, models need to be further defined, adapted, and researched for an effective application in the European context where healthcare systems and cultural diversity of the population are different [21, 23].

Empirical measurement of cultural competence is currently conceptualised as a multidimensional construct that consists of distinct, yet related, factors. Most instruments were thereby developed for a particular group of HCPs (e.g. physicians or nurses) [7, 8]. According to several reviews [24, 25], the four most frequently cited instruments include the Multicultural Counselling Inventory (MCI) [26], the Cross-Cultural Counselling Inventory-Revised (CCCI-R) [27], the Multicultural Awareness-Knowledge-and-Skills Survey (MAKSS, MAKSS-CE-R) [28, 29], and the Multicultural Counselling Knowledge and Awareness Scale (MCAS-B, MCKAS) [30, 31]. All of these instruments use a brief format, multi-factorial design and were developed in the United States for the counselling profession. All except the CCCI-R, an observer-rated measure, are self-report instruments. In general, these instruments are based on Sue et al.’s [18] model and primarily assess the level of awareness, knowledge and skills counsellors possess in working with diverse clientele in a culturally sensitive manner.

Despite considerable advances in cultural competence research, several limitations of current instruments have been noted. One limitation is caused by the insufficient amount of psychometric data available. Recent reviews claim that additional factor analytic and validation studies are needed to improve the overall validity and utility of existing instruments [8, 9, 20, 32]. Although the MCKAS, MCI and CCCI-R were conceptually rooted in the tripartite model proposed by Sue et al. [18], factor analytic studies did not support a three-dimensional structure [24, 33]. For instance, the MCI revealed a four-factor model (accounted for 36% of variance) with unknown test-retest stability [24, 26], the MCKAS showed a two-factor model (accounted for 32% of variance) with questionable criterion validity [31], and the factor structure of the CCCI-R demonstrated both a one-factor and three-factor solution [24]. Other potential concerns with existing instruments include high correlations between dissimilar subscales within an instrument, a lack of clarity of constructs and limitations related to self-report measures (e.g. social desirability) [20].

Currently, no instrument is available to assess cultural competence of different HCPs within a German healthcare context. Thus, this study aimed to 1) identify the core components of cultural competence by incorporating the perspectives of practising HCPs and experts, 2) develop a self-report instrument to assess cultural competence of different HCPs and 3) to evaluate the psychometric properties of the new instrument. It was furthermore hypothesized that this instrument’s items would extend the three-dimensional model underlying the most frequently cited instruments.

## Methods

The development and psychometric evaluation of the cross-cultural competence instrument for the healthcare profession (CCCHP) involved a systematic process [34–37] and proceeded in two phases with ten steps: 1) the instrument development phase including six steps, and 2) the psychometric evaluation phase including four steps (**S1 Fig**). Methods and results for each phase will be presented sequentially. A detailed description of the methods is presented as supporting information (**S1 Methods**).

### Phase 1: Instrument development

In line with Lynn [35] and Liu et al. [37], instrument content was established by a review of the cultural competence literature and existing instruments, an expert survey and interviews with HCPs. Subsequently, items were generated to constitute a preliminary instrument.

Step 1 began with a narrative literature review which aimed to 1) identify conceptual models and existing instruments of cultural competence within a healthcare setting, 2) identify the construct and content domains distinguished, as well as 3) to locate items in existing instruments. Computerized searches of 12 bibliographic databases, Google Scholar and hand searches yielded more than 900 studies.

More than 120 peer-reviewed articles on cultural competence models were identified. Eventually, 14 articles representing unique cultural competence models were retrieved. The most common components of the identified models were cognitive and behavioural components, with few addressing contextual elements. Cultural competence models were later used to guide the development of the CCCHP model.

The database and hand searches yielded 135 citations on cultural competence assessment instruments. For each instrument located, information on the instrument’s name, target population, number of items, dimensions and response scale were retrieved. Twenty-nine instruments were fully available, whereof the vast majority were self-report forms (93%), originated from the United States (86%) and were developed for the field of nursing (21%), counselling (28%) or healthcare (28%). The majority focused on cultural skills, awareness, and knowledge, whereas some also measured one or more of the following: multicultural counselling relationship, cultural desire, cultural encounters, flexibility/openness, emotional resilience, and cultural sensitivity. Two researchers assessed the instruments separately investigating 1) their purpose (designed to evaluate cultural competence of individual HCPs), 2) relevance to healthcare practice in Germany, 3) psychometric properties, 4) conceptualisation of cultural competence, and 5) items used to represent the domains. The researchers judged the appropriateness of each instrument on a 3-point rating scale (not relevant to relevant). Disagreements on instrument appraisal were resolved by consensus. Six instruments were rated relevant, 14 were rated somewhat relevant and nine instruments were excluded as they were found to be irrelevant.

Next, an expert survey and interviews with different HCPs were performed. This approach ensured that a broad range of perspectives were included to inform the development of a conceptual model for the CCCHP. Experts and HCPs were asked to 1) define, from their perspective, cultural competence of HCPs, 2) identify the relevant components of cultural competence and 3) suggest items to capture the relevant components. In total, 23 experts (response rate: 18%) participated in an online survey. To explore the perspectives of practising HCPs, twelve semi-structured interviews were conducted with physicians, medical specialists, registered nurses, midwives and psychotherapists from healthcare settings in Hamburg, Germany. These HCPs were purposefully selected, as they represent the target group for the new instrument. Professionals were required to be working with culturally diverse patients. Of the 12 respondents, half had a migration background and half participated in cultural competence training.

Data analyses were carried out separately for the expert survey and HCPs interviews using qualitative content analysis (QCA) [38]. After comparison of the themes in both the HCP and expert group, the researchers determined that the data exhibited common themes. Consequently, themes were combined to achieve a collective perspective. The results of this QCA were the basis for the development of the CCCHP’s cultural competence model.

Sixteen main categories of cultural competence derived from the QCA were grouped into dimensions by the research team. These categories were structured into five dimensions: attitudes, knowledge, awareness/self-reflection, motivation/emotion, and skills. These dimensions were further classified into the three domains: cognitive, behavioural, and affective (**S1 Fig Step 1**).

In Step 2, items were generated based on suggestions of the experts and HCPs as well as the 20 cultural competence instruments rated as relevant or somewhat relevant. Suggestions provided by experts and HCPs guided the selection of items from existing instruments. An initial item pool of 384 items was generated, based on the instruments (81%), items from experts (6%) and from HCPs (13%). Only individual items were included from existing instruments; scales or subscale as a whole were not incorporated. The item pool was systematically reduced in four assessment rounds by three researchers. The remaining 115 items were translated and language adaptations of items were made to suit the German healthcare system usage.

It has been stressed that self-report instruments of cultural competence may be susceptible to social desirability [39]. To identify individual cultural competence (cc) items that are prone to elicit socially desirable responding, six items (SD markers) of the Social-Desirability Scale-17 (SDS-17) [40] were found to be appropriate in terms of content (face validity) and were modified (i.e. culturally adjusted) for inclusion in the CCCHP.

A 5-point Likert Scale (from strongly agree to strongly disagree) response format with a ‘no answer possible option’ was selected for the 115 cc items and 6 SD items. This scale was chosen as it allowed a neutral midpoint for respondents who may truly be indifferent with their degree of agreement regarding an item. A great number of responses in the neutral midpoint of an item would indicate that this item needs to be rephrased to better differentiate.

In Step 3, the draft 115-item instrument and the 6 SD items were administered to a convenience sample [41] of researchers and psychology students (n = 13). The respondents judged how representative the individual items were of the construct content domain [42], evaluated item clarity, checked for redundancies and suggested revisions for item/instrument construction. This work yielded an 85-item instrument (plus 6 SD items) that was provided to experts for validation.

Step 4 served to establish face validity of the CCCHP’s content. An expert panel (n = 5, response rate: 33%) was used to evaluate whether the items measure what they were intended to measure [43]. Quantitatively, experts were asked to judge 1) the relevance of each item to measure the respective dimension [42], and 2) if the item was correctly classified to capture the respective dimension. Qualitatively, comments on the clarity and meaning of item construction and wording were also elicited, including suggestions for modifications and refinement [42, 44].

Step 5 yielded the 59-item self-report CCCHP which is distributed across five dimensions. Five of these items originated from existing instruments. Six SD items were embedded to identify individual cc items that contained socially desirable content. Each item was measured with a 5-point Likert-type response scale from 1 = strongly agree to 5 = strongly disagree and a ‘no answer possible’ option. Sum scores are calculated for each subscale. In Step 6, two online surveys of the CCCHP (only differing in background information) were established for the groups of respondents in the psychometric survey. The CCCHP -59 as well as the 6 SD items were presented in a forced-choice response format. To examine usability, the online surveys were pre-tested by 12 psychology students.

### Phase 2: Psychometric evaluation of the CCCHP

#### Study participants

After minor revisions, the 59-item CCCHP was tested for its psychometric properties with two prospective HCP groups: medical students (MS; ≥5^th^ semester and thus in the clinical part of their studies and practical year) and psychologists in advanced psychotherapeutic training (PiA). Both groups were selected as they constitute the later target group for the CCCHP and have patient contact and clinical experience, at this stage of their professional development. In addition to the CCCHP, socio-demographic (e.g. migration background [45]) and occupational/educational information were collected anonymously for the psychometric survey.

An email invitation explaining the purpose of the study was sent to student representatives and dean’s offices of Medical Universities throughout Germany (n = 34). To reach PiAs, requests were sent to different networks and to all 180 PiA training institutions in Germany. As an incentive, participants were offered the opportunity to register for a lottery to win one of ten €20 gift certificates.

#### Data analysis

Analyses focused on testing 1) the suitability of the data set, 2) the instrument’s dimensionality (PCA; principal component analysis), 3) reliability, and 4) discriminating power (known-groups technique) [41] undertaken with the Statistical Package for Social Sciences version 18.0 (SPSS® Inc., Chicago, IL, USA).

In Step 1, pre-analysis checks were executed to ensure the suitability of the data set for factor analysis [46]. The checks included determining the stability of the emerging factor structure, adequacy of sample size, item scaling, skewness and kurtosis of item distribution and the appropriateness of the correlation matrix. To evaluate the instruments practicability and acceptance, response rates, time to complete and feedback from respondents were analysed. In the statistical analyses, the ‘no answer possible’ option (forced-choice responses) was coded as missing. Missing values of ≥30% in any item or respondent were considered to be inadequate for inclusion and were thus eliminated from further analysis. Concerning each item’s distribution, the maximum acceptable proportion of floor and ceiling effects among items as well as subscales was <80% [47].

In Step 2, PCA were performed on the CCCHP-59 (plus 6 SD items) with orthogonal rotation (varimax with Kaiser normalisation) to determine the dimensionality of the instrument. Prior to PCA, the suitability of the data for factor analysis was assessed by the application of Bartlett’s test of sphericity [48] and the Kaiser-Meyer-Olkin (KMO) measure of sampling adequacy [49, 50]. Criteria for extraction of CCCHP components as proposed by Kline [51] and others [52, 53] included: 1) eigenvalues >1, 2) Cattell’s [54] scree test, 3) interpretability of the solution, using a component loading cut-off of .39 and no cross-loadings greater than or equal to .40, and 4) percentages of explained variance (minimum of 5% reported variance) per component. Moreover, methodologists have recommended that at least three to five items represent each component [55]. In this study, the minimum amount of items loading on each component was set to four. Items were excluded if they met one of the following criteria: 1) weak loadings (failing to load above .39 on any component), 2) general loadings of .40 on more than one component, 3) ≥30% of responses were missing or 4) ≥80% of item responses were the same (floor/ceiling effect).

In Step 3, internal consistency reliability was assessed for the CCCHP using Cronbach’s α to address the homogeneity of items in a scale. The criteria for Cronbachs α as described by DeVellis [56], were applied for the interpretation of results (α of >.70). The SD items were included in the PCA 1) to evaluate the reliability and factorial validity of the reduced and modified SD scale and 2) to identify cc items with qualities of SD items.

In Step 4, in addition to PCA, construct validity of the CCCHP was assessed by using the known-groups technique [41]. Three subgroups were predefined based on theoretically expected differences in cultural competence. On the assumption that having a migration background, frequent cross-cultural encounters and participating in cross-cultural competence training would lead to higher scores on the CCCHP subscales, mean scores were calculated. Higher mean scores indicate a higher motivation, more positive attitudes and emotions, greater frequency of using culturally competent skills, and higher levels of knowledge. Higher mean scores on the SD subscale indicate a higher tendency to provide socially desirable responses. The independent sample t-test was used to analyse differences between group means on each CCCHP subscale and the SD subscale [37]. The significance level was set at p < .05.

#### Ethics Statement

This study is part of the international research project on ‘Mental Health and Migration’ (www.segemi.de), approved by the Ethics Committee of the Hamburg Chamber of Psychotherapists, Germany. All participants received written information regarding the study according to the principles outlined in the Declaration of Helsinki. In the online psychometric survey, the written information contained a passage informing the participants that they declare their consent by completing the CCCHP-59.

## Results

### Study participants

A convenience sample of 409 medical students (MS) and 336 PiA participated. One-fifth (21.3%) of the MS and one-third (30.9%) of the PiA in this study had a migration background. A foreign nationality was recorded for 6.9% of the MS and 8.7% of the PiA. Almost all respondents (MS: 99.0% vs. PiA: 93.5%) stated to have patient contact, although the length of contact differed significantly between the groups. Regarding cross-cultural competence training, 9.8% of the MS and 22% of the PiA reported participating in training activities. There was a significant difference between MS and PiA in demographic characteristics, including age, gender, migration background, staying abroad and participation in cultural competence training (p < .05). Demographic characteristics for the MS and PiA sample are shown in **Table 1**.

**Table 1:** Demographic characteristics of the medical students and PiA sample.

	Total sample (n = 745) n (%)		Medical students (n = 409) n (%)		PiA (n = 336) n (%)	
Age (in years), mean [SD]	29.4	[7.3]	25.4	[3.2]	34.3	[7.8]
Gender						
Male	129	(17.3%)	93	(22.7%)	36	(10.7%)
Female	616	(82.7%)	316	(77.3%)	300	(89.3%)
German nationality	681	(91.4%)	377	(93.1%)	304	(91.3%)
Foreign nationality	57	(7.7%)	28	(6.9%)	29	(8.7%)
Migration background	191	(25.6%)	87	(21.3%)	104	(30.9%)
1-sided migrant	63	(8.5%)	31	(7.6%)	32	(9.5%)
2-sided migrant	109	(14.6%)	48	(11.7%)	61	(18.2%)
Patient contact: Yes	719	(96.5%)	405	(99.0%)	314	(93.5%)
< 2 months/year	224	(30.1%)	185	(45.7%)	39	(12.4%)
2–6 months/year	118	(15.8%)	89	(22.0%)	29	(9.2%)
> 6 months/year	368	(49.4%)	127	(31.4%)	241	(76.8%)
Frequent cross-cultural contact						
Own family/relatives	234	(31.4%)	120	(29.3%)	114	(34.3%)
Study/work	545	(73.2%)	319	(78.0%)	226	(67.3%)
Neighbourhood	280	(37.6%)	165	(40.3%)	115	(34.6%)
Friends/acquaintance	444	(59.6%)	261	(63.8%)	183	(54.5%)
Stay abroad						
> 2 months (within past 5 years)	248	(33.3%)	157	(38.4%)	91	(27.1%)
Cultural competence training						
Participated	114	(15.3%)	40	(9.8%)	74	(22.0%)
Rated as (very) helpful	104	(14.0%)	38	(95.0%)	66	(89.1%)
Study progress						
Pre-clinical			7	(1.7%)	N/A	
Clinical (≥ 5^th^ semester)			333	(81.4%)	N/A	
Practical year			69	(16.9%)	N/A	
Clinical traineeships completed						
1			90	(14.2%)	N/A	
2			59	(22.0%)	N/A	
3			57	(13.9%)	N/A	
4			66	(16.1%)	N/A	
Training specialisation						
Behavioural therapy (VT)			N/A		239	(71.1%)
Psychodynamic Psychotherapy (TP)			N/A		60	(17.9%)
Psychoanalysis (PA)			N/A		36	(10.7%)

PiA, Psychologists in advanced psychotherapeutic training; SD, Standard deviation; Migration background, includes I) individuals who have migrated themselves, II) someone with 1 parent immigrated, or III) someone with both parents immigrated and/or do not have German citizenship [45]; 1-sided migrant, if only 1 parent immigrated [57]; 2-sided migrant, if both parents, or the child and 1 parent, immigrated [57]; N/A, Not Applicable

In Step 1, the pre-analysis checks proposed by Ferguson and Cox [46] were applied to the CCCHP-59 (plus 6 SD items). The sample size was adequate, with an acceptable minimum ratio of participants to items (N/p ratio) of 11:1 [58, 59]. Furthermore, the ratio of participants to expected factors (N/m ratio) exceeded the acceptable minimum with 123:1. The minimum ratio of items to expected factors (p/m ratio) was achieved with a ratio of 10:1 [60].

All except two CCCHP items (#47, #57) showed an acceptance rate above 70%. Mean completion time for the total survey (including background information) was 16.5 minutes (SD: ± 5.5 minutes). On average, 61 of the 65 items (96.2%) were completed. For 51 (78.5%) of the 65-items, the percentage of missing data was lower than 5%. The initial data analysis led to the exclusion of two items (#47, #57) with unacceptable levels of missing data (≥30%), and seven respondents omitting more than 19 items (≥30%). Missing values were excluded pairwise in the PCA.

According to Gorsuch [61], exploratory factor analysis appears to be relatively robust against violations of normality, and no problems with excessive item skewness (absolute values above 3) or kurtosis (absolute values above 10) were found [62].

In Step 2, the inspection of the correlation matrix revealed the presence of coefficients ≥.30 and indicated the absence of multicollinearity. The KMO measure exceeded the recommended value of 0.60 [50] with 0.888. Bartlett’s test of sphericity [48] was statistically significant, supporting the adequacy of the data and sampling for factor analysis (x^2^ = 9227.795, df = 1953, p < .001). An initial unrotated PCA performed on the 57 CCCHP items and the SD items revealed the presence of 15 components with eigenvalues >1. Together these 15 components accounted for 54.4% of the total variance. On the basis of Cattell’s [54] scree test, an approximate solution of six components was indicated. The extraction of four, five, six, and seven components was considered. Both orthogonal and oblique rotations were conducted. Absolute correlations between components with the oblique solution ranged from -.045 to -.367 with only 1 of the 15 correlations greater than .30. Tabachnick and Fidell [53] suggested a cut-off of .30 to adopt an oblique rotation. Given that most of the correlations between components were relatively small, the orthogonal (varimax) rotation was deemed to be appropriate as it provided the clearest and most interpretable solution.

In line with an iterative process described in detail previously [52, 63], items with cross-loadings (of .40 on more than one component) and weak loadings (failing to load above .39 on any component) were excluded one at a time from the initial unrotated and subsequent rotated PCA. Following this item reduction process, a re-run of the PCA using orthogonal (varimax) rotation led to a six component solution. In order to reach purity of subscales and meaningfulness of content, 31 items were excluded. Thus, a final 32-item solution, accounting for 50.2% of the variance was identified. Over 50% of the items that failed to meet the eligibility criteria for the final 32-item solution (CCCHP-27 plus 5-item SD subscale) reflected cognitive aspects (knowledge and awareness) of cultural competence. The six-component orthogonal solution was judged to yield the most interpretable solution (**Table 2**). None of the 27 cc items included were literally translated from existing questionnaires.

**Table 2:** Principal component analysis with varimax rotation. Final six-component solution with Cronbach’s α of each component.

Item No.*	Rotated Component Loadings						
	Motivation	Attitudes	Skills	Social desirability	Emotions/ Empathy	Knowledge/Awareness	Commu-nalities
42	**0,725**	0,050	-0,062	0,192	0,064	0,099	0.583
12	**0,667**	0,190	-0,076	0,162	0,013	0,162	0.539
64	**0,658**	0,270	0,209	0,037	0,130	0,020	0.567
1	**0,655**	0,304	0,042	0,169	0,076	-0,028	0.558
17	**0,651**	0,149	0,307	0,022	0,022	-0,085	0.549
29	**0,573**	0,050	0,302	-0,080	-0,020	0,062	0.432
10	**0,549**	0,019	0,282	-0,083	0,154	0,203	0.453
38	**0,468**	0,329	0,279	-0,006	-0,281	-0,005	0.484
58	**0,435**	0,253	0,389	0,100	0,004	0,087	0.422
8	0,115	**0,765**	0,141	0,004	0,131	0,051	0.638
43	0,205	**0,744**	0,109	-0,015	0,077	0,071	0.618
60	0,249	**0,685**	0,112	-0,005	0,032	0,106	0.555
21	0,213	**0,667**	0,003	-0,026	0,158	0,053	0.519
50	0,085	0,063	**0,695**	0,343	0,036	0,019	0.614
5	0,200	0,130	**0,613**	-0,122	0,102	-0,082	0.464
44	0,031	-0,022	**0,599**	0,324	0,051	0,057	0.471
51	0,258	0,067	**0,551**	-0,099	-0,032	0,243	0.444
53	0,241	0,154	**0,493**	0,098	0,126	0,232	0.405
61 SD	0,157	-0,134	-0,037	**0,686**	0,096	0,048	0.526
36 SD	0,011	0,017	0,102	**0,686**	0,148	-0,098	0.513
28 SD	0,034	0,019	0,172	**0,685**	0,114	0,017	0.514
19	0,092	0,003	0,011	**0,584**	0,197	-0,200	0.428
31 SD	0,066	0,198	0,363	**0,438**	0,117	-0,150	0.403
55	0,071	0,211	-0,064	0,119	**0,694**	0,030	0.551
18	-0,006	0,103	-0,003	0,164	**0,683**	-0,113	0.517
63	0,018	0,252	0,236	0,145	**0,620**	-0,057	0.528
26	0,041	-0,166	0,161	0,138	**0,574**	-0,053	0.407
48	0,320	0,217	-0,033	0,345	**0,456**	-0,106	0.489
30	0,093	0,000	0,186	-0,092	-0,009	**0,713**	0.560
9	0,023	0,135	-0,091	-0,008	-0,095	**0,696**	0.520
11	0,087	0,084	0,071	-0,352	-0,139	**0,543**	0.457
25	0,379	0,054	0,192	0,036	-0,022	**0,405**	0.349
Eigenvalues after rotation	3.937	2.783	2.703	2.651	2.205	1.799	
% explained variance	12.302	8.697	8.446	8.285	6.890	5.623	
Cumulative %	12.302	20.999	29.444	37.729	44.620	50.243	
Cronbach’s α	.836	.780	.684	.714	.694	.543	.869

* For descriptions of the items see Table 3.

The bold figures signify the highest component loading for each item.

### Interpretation of components

The rationale used for naming the six components was partly guided by the recommendations of Ferguson and Cox [46] and Cattell [60], to use marker items to drive the process of labelling each component which emerged from the analysis. Furthermore, the hypothesized components were defined and named before the analysis, and therefore did not rely on post hoc explanations (**Table 3**).

**Table 3:** Components of the CCCHP-27 with corresponding items (*English*).

Component	Item No.	Sample Items*
Cross-Cultural Motivation/Curiosity (CC-MC)	42	I consider it an enrichment to have friendships with people from different cultural backgrounds.
	12	Cultural diversity is also an enrichment.
	64	I find it exciting to treat patients with a migration background.
	1	I consider working in a cross-cultural team an enrichment.
	17	I enjoy talking to people who have migrated to [country concerned] about their experiences here.
	29	The interaction with people from other cultural backgrounds helps me reflect upon my own cultural background.
	10	By communicating with patients with a migration background I can learn about different cultural orientations.
	38	I would like to make use of training, advising and educational offers, in order to improve my understanding of patients with a migration background.
	58	It is important for me to treat patients according to their cultural needs and individual values.
Cross-Cultural Attitudes (CC-A)	8^a^	I find it an imposition, when people who migrated to [country concerned] a long time ago, cannot speak [language concerned] properly.
	43^a^	People who migrated to [country concerned] should adapt to society, not the other way around.
	60^a^	Institutions and the public pay too much attention to the special wishes of migrants.
	21^a^	I have the impression that migrants often assume discrimination, when in fact general rules are simply being enforced.
Cross-Cultural Skills (CC-S)	50	With patients who do not understand [language concerned] very well, I take more time to explain the treatment options to them.
	5	In order to achieve the agreed treatment goal, I ask patients with a migration background what they need in terms of support.
	44	With patients who do not understand [language concerned] very well, I allow for more time to explain the treatment options to them.
	51	Culturally specific factors of people (e.g. values, behaviour norms, beliefs) influence their understanding of disease significantly, and should therefore be assessed and taken into consideration by healthcare professionals.
	53	I consider the values of patients in relation to family, religion, etc., if they seem relevant for the treatment.
Cross-Cultural Emotions/Empathy (CC-EE)	55^a^	In my professional interaction with patients with a migration background, I often feel unsure, angry and frustrated.
	18^a^	I often find it difficult to relate to the elaborations of my patients, when their socio-cultural background is quite different from my own.
	63^a^	I get impatient when I cannot make myself understood with patients with a migration background.
	26^a^	I find it difficult to speak slowly in lay language with people who struggle to understand my instructions.
	48^a^	I prefer treating patients from my own cultural background, than those who seem foreign to me.
Cross-Cultural Knowledge/Awareness (CC-KA)	30^a^	The disease concepts of patients with a migration background are not relevant for treatment success.
	9^a^	Within the migrant population, there are hardly any differences in terms of health opportunities and disease risks.
	11^a^	My professional perception, assessment, and behaviour remain untouched by my cultural imprinting.
	25	The migration experience is a critical life event and can be accompanied by psychosocial stress and health burden.

*Items were translated into English [64]. See S1 File for the original German version of the 27-item CCCHP.

^a^ Items are presented in reverse to prevent tendencies towards the response set.

#### Component 1 (Cross-Cultural Motivation/Curiosity (CC-MC))

The first component (Cronbach α .84, M = 4.14, SD = 0.560) included nine items (#42, #12, #64, #1, #17, #29, #10, #38, #58) with moderately high loadings between .435 and .725, accounting for 12.3% of the total variance. The items referred to HCPs’ motivation to provide culturally responsive care, to their curiosity to engage in cross-cultural encounters and a wish to enrich their understanding in working with culturally different populations. Seven of these items were originally hypothesised to reflect professionals’ motivation, while two items represent HCPs’ openness towards cultural diversity.

#### Component 2 (Cross-Cultural Attitudes (CC-A))

Four items (#8, #43, #60, #21) with relatively high and specific loadings between .667 and .765 loaded on component 2 (Cronbach α .780, M = 3.11, SD = 0.760), accounting for 8.7% of the total variance. All four items were originally hypothesised to represent attitudes such as tolerance, valuing and respecting differences, and having a positive orientation towards other cultures and cultural diversity.

#### Component 3 (Cross-Cultural Skills (CC-S))

The third component (Cronbach α .684, M = 3.90, SD = 0.566) included five items (#50, #5, #44, #51, #53) with loadings between .493 and .695 accounting for 8.5% of the total variance. This component considered HCPs’ adaptability in meeting the (cultural) needs of their patients, professionals’ communicative competence and to make time for their patients. Four items were originally hypothesised to reflect culturally competent skills, whereas one item represented knowledge to incorporate patients’ concepts of health and illness in the course of the treatment.

#### Component 5 (Cross-Cultural Emotions/Empathy (CC-EE))

The fifth component (Cronbach α .694, M = 3.50, SD = 0.648) consisted of five items (#55, #18, #63, #26, #48) with loadings ranging from .456 to .694, accounting for 6.9% of the total variance. The items referred to feelings and emotional reactions towards diversity, with being comfortable with difficulties arising in cross-cultural encounters and with being multicultural empathic. Two of these items were originally hypothesised to reflect emotions (e.g. to feel comfortable and xenophilous), whereas three items reflected skills (e.g. empathy, sensitivity and patience).

#### Component 6 (Cross-Cultural Knowledge/Awareness (CC-KA))

Four items (#30, #9, #11, #25) loaded between .405 and .713 on the sixth component (Cronbach α .543, M = 4.26, SD = 0.561) and accounted for 5.6% of the total variance. The items referred to cultural and migration-specific knowledge, to an understanding of concepts of illness and health, and to an awareness of one’s own perceptions and values. Three of these items were originally assumed to represent knowledge and one item was associated with professionals’ self-awareness.

#### Component 4 (Social Desirability (SD))

The fourth component (Cronbach α .714, M = 3.96, SD = 0.554) represented the culturally adjusted social desirability scale, as four of the six SD items (#61, #36, #28, #31) were included. In total, five items with moderately high loadings between .438 and .686 represented the SD scale and accounted for 8.3% of the total variance. One other item (#19) loaded on the SD scale. The item was originally hypothesized to reflect cross-cultural attitudes.

Descriptive statistics for each subscale were derived. Acceptance, item means and standard deviations as well as skewness and kurtosis for items included in the final scale are shown in **Table 4**. The item analyses of the final subscales showed positive endorsement of the majority of items and the subscales were positively skewed. Positive endorsement rates were highest for the motivation subscale, especially for two items (#42, #12), and the knowledge/awareness subscale (#30, #25). No items showed floor effects in any of the subscales.

**Table 4:** Item/Subscale characteristics.

Item No.*	Acceptance (response in %)	Mean (SD) (range 1–5)	Corrected item-total correlation subscales	Ceiling effect (%)	Floor effect (%)	Skew	Kurtosis
***Motivation***						***- 0*.*744***	***0*.*273***
42	99.5	4.58 (0.625)	0.545	64.2	0.1	- 1.527	2.704
12	99.9	4.67 (0.598)	0.536	73.0	0.1	- 1.867	3.613
64	98.5	3.74 (0.954)	0.657	23.3	1.6	- 0.409	- 0.243
1	98.6	4.27 (0.801)	0.606	46.5	0.0	- 0.787	- 0.247
17	99.5	3.86 (0.961)	0.633	29.1	1.2	- 0.531	- 0.284
29	99.7	4.02 (0.978)	0.527	36.3	2.0	- 0.965	0.561
10	99.7	4.43 (0.706)	0.489	53.4	0.1	- 1.202	1.518
38	99.3	3.78 (1.119)	0.500	30.9	3.4	- 0.683	- 0.403
58	98.4	3.90 (0.825)	0.515	24.1	0.1	- 0.358	- 0.371
***Attitudes***						***- 0*.*094***	***-0*.*160***
8	99.9	2.69 (1.097)	0.609	5.7	14.9	0.211	- 0.625
43	98.8	2.78 (0.943)	0.630	5.6	7.2	0.326	0.133
60	95.3	3.81 (0.906)	0.560	21.3	1.6	- 0.660	0.377
21	94.0	3.91 (0.938)	0.548	5.7	3.4	- 0.205	- 0.393
***Skills***						***- 0*.*317***	***-0*.*030***
50	92.1	3.64 (0.822)	0.577	11.5	0.3	- 0.333	- 0.209
5	91.9	3.48 (1.058)	0.373	15.7	3.3	- 0.370	- 0.551
44	88.8	3.67 (0.899)	0.454	13.4	1.4	- 0.606	0.159
51	99.5	4.31 (0.720)	0.369	44.6	0.1	- 0.860	0.603
53	97.8	4.28 (0.650)	0.479	37.1	0.0	- 0.563	0.291
***Social desirability***						***- 0*.*431***	***0*.*080***
61 SD	97.0	4.27 (0.833)	0.441	45.8	0.3	- 1.070	0.753
36 SD	90.8	3.64 (0.810)	0.538	11.4	0.8	- 0.337	0.184
28 SD	98.6	3.89 (0.776)	0.553	19.8	0.4	- 0.513	0.385
19	96.7	4.01 (0.914)	0.446	30.2	1.2	- 0.961	0.744
31 SD	98.4	4.00 (0.699)	0.395	22.9	0.0	- 0.233	- 0.314
***Emotions/Empathy***						***- 0*.*219***	***-0*.*269***
55	92.8	3.35 (0.967)	0.489	10.6	2.6	- 0.175	- 0.388
18	95.4	3.36 (0.883)	0.477	8.5	1.5	- 0.117	- 0.238
63	97.4	3.32(0.939)	0.509	8.4	2.0	- 0.204	- 0.505
26	99.2	3.99 (0.897)	0.346	30.8	0.7	- 0.771	0.234
48	98.1	3.48 (1.127)	0.442	21.1	3.9	- 0.293	- 0.798
**Knowledge/Awareness**						***- 0*.*859***	***0*.*550***
30	98.4	4.51 (0.742)	0.421	61.0	0.8	- 1.849	4.297
9	96.6	4.11 (0.834)	0.339	32.9	0.9	- 1.045	1.411
11	99.2	3.77 (1.142)	0.362	31.6	3.9	- 0.695	- 0.419
25	99.6	4.64 (0.596)	0.250	69.2	0.0	-1.682	2.814

* Total: 32 items after principal component analysis (PCA); %Ceiling/Floor effect = the percentage of scores at the extremes of the scaling range; Higher values equal higher cross-cultural competence.

In Step 3, Cronbach’s α were calculated for the total sample and subscale separately. Cronbach’s α for the overall scale was .869 and subscale α varied between .543 and .836. The internal consistency coefficient for the CC-KA subscale was low (α .543).

Six SD items were included to identify CCCHP items with socially desirable content. The mean response (range 1–5) to SD items was 3.82 (SD = 0.537). A higher mean response reflected a higher tendency to provide answers in a socially desirable manner. Spearman’s coefficient correlations (r_s_) between individual CCCHP items and the SD items as well as correlations between the CCCHP subscales and the a priori defined 6-item SD scale were examined. Correlations of the attitudes and knowledge/awareness subscales with the SD items and SD subscale were trivial in terms of magnitude with a majority being statistically non-significant. However, there was a significant relationship between four CCCHP items (#19, #48, #50, #63) and the six SD items. The absolute values of correlations with these items ranged from .12 to .36 and the average correlation was .25 (all ps < .001). The absolute values between the 6-item SD scale (Cronbach α .722) and the CCCHP subscales ranged from .11 to .45 and the average correlation was .25. The SD scales’ highest correlation was with the CC-EE subscale (r_s_ = .45, p < .000).

These results indicated that although the CC-A and CC-KA subscales appeared to be unrelated to social desirability, the CC-EE, CC-MC and CC-S subscales were to some degree related to a tendency to respond in a socially desirable manner.

In Step 4, the known-groups technique was carried out to provide further evidence of construct validity. Mean subscale scores (range 1–5) of the CCCHP items were calculated with higher scores indicating higher cultural competence, whereas a higher score on the SD subscale indicating a higher tendency to provide socially desirable responses. Subscale mean scores of respondents having theoretically expected higher cultural competence were in over 65% of the cases statistically significantly higher than those of respondents with theoretically expected lower cultural competence (p < .05) (**Table 5**). Respondents with a migration background had on three of the five cc subscales statistically significantly higher mean scores than respondents without a migration background. The mean scores of respondents with frequent cross-cultural encounters through family, neighbourhood, friends or study/work were in two of the five cc subscales significantly lower than of respondents with little cross-cultural encounters in these areas. As hypothesized, mean scores of respondents who reported receiving previous cross-cultural competence training were significantly higher on four of the five cc subscales than those who reported no training.

**Table 5:** Known-groups technique.

Group	n	Mean score (SD) Group A: Theoretically expected higher cultural competence		Mean score (SD) Group B: Theoretically expected lower cultural competence		Significance		
						t	df	p
Migration background (A)	191	CC-MC:	4.23 (0.51)	CC-MC:	4.11 (0.57)	-2.70	736	.007*
vs. no migration background (B)	547	CC-A:	3.23 (0.74)	CC-A:	3.07 (0.76)	-2.52	736	.012*
		CC-S:	3.92 (0.58)	CC-S:	3.89 (0.56)	-0.46	736	.644
		SD:	4.03 (0.61)	SD:	3.94 (0.53)	-1.86	294	.063
		CC-EE:	3.69 (0.65)	CC-EE:	3.44 (0.63)	-4.63	735	.000*
		CC-KA:	4.20 (0.62)	CC-KA:	4.28 (0.53)	1.71	296	.089
Frequent cc contact: total (A)	94	CC-MC:	4.31 (0.50)	CC-MC:	4.12 (0.56)	-3.10	736	.002*
vs. little cc contact: total (B)	644	CC-A:	3.24 (0.78)	CC-A:	3.09 (0.75)	-1.80	736	.073
		CC-S:	3.98 (0.51)	CC-S:	3.89 (0.57)	-1.48	736	.140
		SD:	4.06 (0.60)	SD:	3.95 (0.55)	-1.85	736	.064
		CC-EE:	3.78 (0.60)	CC-EE:	3.46 (0.64)	-4.52	735	.000*
		CC-KA:	4.20 (0.61)	CC-KA:	4.27 (0.55)	1.12	736	.262
Participated in cross-cultural training (A)	112	CC-MC:	4.44 (0.41)	CC-MC:	4.09 (0.57)	7.86	196	.000*
vs. no participation in cc-training (B)	624	CC-A:	3.35 (0.67)	CC-A:	3.07 (0.77)	3.54	734	.000*
		CC-S:	4.12 (0.48)	CC-S:	3.86 (0.57)	4.52	734	.000*
		SD:	3.87 (0.57)	SD:	3.98 (0.55)	-2.06	734	.040*
		CC-EE:	3.52 (0.54)	CC-EE:	3.50 (0.67)	.41	177	.685
		CC-KA:	4.41 (0.56)	CC-KA:	4.24 (0.55)	2.98	734	.003*

CC-MC: Cross-Cultural Motivation/Curiosity; CC-A: Cross-Cultural Awareness; CC-S: Cross-Cultural Skills; SD: Social Desirability; CC-EE: Cross-Cultural Emotions/Empathy; CC-KA: Cross-Cultural Knowledge/Awareness (mean: range 1–5).

*statistically significant with p < .05. Higher values equal higher cross-cultural competence.

To summarize, for the CC-MC subscale all group comparisons were statistically significantly different, whereas for the CC-EE and CC-A subscales only two of the three comparisons were statistically significantly different. In contrast, only one group comparison in the CC-S and CC-KA subscales was statistically significantly different. Apparently, these two subscales have less discriminatory power for group differences.

## Discussion

One of the major challenges in current research on cultural competence is the limited amount of validated conceptual models and assessment instruments suitable for the German healthcare context. The CCCHP developed for the assessment of cultural competence in HCPs, was shown to have good content and construct validity and modest reliability.

*Content validity* is of utmost importance because it ensures congruence between the research objective and the data gathering instrument [37, 65]. The evidence supporting the content validity of the CCCHP was based, firstly, on a literature review of cultural competence models and assessment instruments and, secondly, on an expert survey and interviews with HCPs. Sixteen core components of cultural competence grouped into a five-dimensional model: (attitudes, knowledge, awareness/self-reflection, motivation/emotion, and skills) were identified.

Attitudes, knowledge and skills are also part of the tripartite model proposed by Sue et al. [18] and are emphasised in most assessment instruments [32]. Cultural desire and awareness have been acknowledged by Campinha-Bacote [12] and others [10, 66] as necessary for the assessment of cultural competence. However, emotions and self-reflection as part of the CCCHP’s conceptual model appear to tap relatively unique dimensions of cultural competence. HCPs are encouraged to explore their own thoughts and emotions and how these influence their work with patients. Collins and Pieterse [67] highlighted unmasking the subconscious, namely, automatic responses that guide and frame individual’s feelings and cultural interactions as a key ingredient of multicultural competency. Furthermore, active engagement in self-reflective processes was suggested to have great potential to increase counsellors’ multicultural competence [68]. Accordingly, the conceptualisation of the CCCHP extends the three-dimensional model underlying the most frequently cited instruments.

Additional strengths of this study include the explicit incorporation of perspectives of HCPs and experts from the beginning of the development process as well as the use of various sources to generate items for inclusion in the initial item pool [69]. This approach ensured that the CCCHP covered aspects, which are considered important to professionals’ everyday practice, namely to provide appropriate care to diverse patient populations. The need to progress from a theorists’ conceptualisation of cultural competence to a more practice-orientated conceptualisation was also highlighted in the literature [70]. Several phases of item reduction ensured that only the most relevant items were included in the final instrument. The relevance of feedback obtained from HCPs and experts in formulating and selecting the content of items can be illustrated by the final set of items. While 81% of the initial item pool came from existing instruments, the 59-item version contained only 5 items, while in the final 27-item version, no item originated from existing instruments.

*Construct validity* is the extent to which an instrument measures the theoretical construct it is intended to measure [71]. Construct validity of the CCCHP -59 was assessed using PCA and the known-groups technique. PCA on the data set led to a 32-item (CCCHP -27 plus 5-item SD subscale) six-component solution with 50.24% of total variance explained.

The results of the PCA indicate that the CCCHP is a multidimensional instrument composed of five dimensions of HCPs’ cultural competence: Cross-Cultural Motivation/Curiosity, Cross-Cultural Attitudes, Cross-Cultural Skills, Cross-Cultural Knowledge/Awareness and Cross-Cultural Emotions/Empathy. Hence, the CCCHP provides an extension of the predominant conceptualisation of cultural competence.

‘Cross-Cultural Motivation/Curiosity’ was a clear and strong component as indicated by the moderate or high loadings of items on this component. At present, few existing measures of cultural competence include this relevant component as an independent dimension [72].

The second component, ‘Cross-Cultural Attitudes’ is in line with Mason [73] and Ponterotto et al.’s [74] endeavour to assess attitudes towards ethnical diversity and multiculturalism. Furthermore, Sue and Zane [75] pointed out that differences in cultural attitudes and beliefs between the professional and patient affect the process and efficacy of the therapy.

The third component ‘Cross-Cultural Skills’ relates to the HCPs’ ability to work with culturally diverse patients and also receives emphasis in the existing literature [14, 26, 29].

The ‘Cross-Cultural Emotions/Empathy’ component is supported by Gerrish, Husband and Mackenzie [76] who recognized emotions, such as ‘an emotional openness to others’ or ‘inner-directed emotions’ as the affective dimension of intercultural communicative competence.

The knowledge and awareness dimensions were originally hypothesized to yield different subscales. However, ‘Cross-Cultural Knowledge/Awareness’ appears to focus on two interrelated areas of HCPs’ cultural competence. This finding is consistent with previous research that suggested knowledge and awareness as inseparable cognitive components of cultural competence [77]. ‘CC-KA’ is consistent with the literature that focuses on professionals’ self-awareness and knowledge about cultural diversity and its implications for practice [14, 26].

A ‘Social Desirability’ subscale was identified. In order to check for the potential influence of social desirability, a number of researchers suggest including scales that are context specific and can be used to statistically control for this bias [19, 39]. One item ‘*I do not differentiate between the patients …*’, which was originally hypothesised to reflect cross-cultural attitudes, loaded on this component. This item may however reflect dependence on the acceptance, and approval of others.

With regard to construct validity, the domains defined a priori in the conceptual model were similar to those indicated by the PCA. This similarity reinforces the validity of the chosen procedure.

To support construct validity, the known-groups technique was applied to test the CCCHP’s ability to discriminate between the predefined groups [41]. The discriminating power of the CCCHP was supported by statistically significantly mean differences between the three predefined groups (p < 0.05). Similar to findings by Doorenbos et al. [77], respondents who had previously participated in cross-cultural competence training, presented statistically significantly higher mean scores than those who had not had this kind of training. Nevertheless, the results of the known-groups technique need to be interpreted in light of the potential influence of social desirability.

In the current study Cronbach’s α was used to estimate the reliabilities of the total scale and of each dimension. The overall scale performed well, with a Cronbach’s α reliability coefficient of .869. However, reliability coefficients of three (.543, .684, .694) of the respective CCCHP subscales were below .70. DeVellis [56] suggested an α of .70 as acceptable for a new instrument. Specifically, the coefficient value for the ‘Cross-Cultural Knowledge/Awareness’ was substantially below the recommended cut-off of .70. To measure ‘CC-KA’ more reliably, a larger pool of suitable items will have to be developed and tested. Further research with practising HCPs will be necessary to enhance the reliability of these scales.

The results of the preliminary analysis on the psychometric properties of the new instrument provide satisfactory evidence with respect to acceptability and feasibility. The higher non-response rate in the skills dimension (range: 0.5–11.2%) could reflect respondents’ lack of clinical experience. In general, however, the overall small proportion of missing values confirmed the acceptability of the CCCHP.

Mean completion time for the total survey was 16.5 minutes (SD ± 5.5 minutes). Nevertheless, feasibility with regard to completion time will be improved due to the reduced number of items in the final version of the instrument. Positive endorsement rates were highest for the motivation/curiosity and knowledge/awareness subscale. In this respect, more differentiated response options would be useful to improve the CCCHP subscales.

### Limitations

This study has several strengths, but also limitations that need to be considered when interpreting the results.

While this study enhances the existing understanding of cultural competence by incorporating aspects considered important by practising HCPs in addition to experts, one limitation of this study is that it does not include the patients’ perspective [78, 79]. Despite a low response rate of HCPs and experts, multiple perspectives could be incorporated to inform the development of the CCCHP’s conceptual model and item pool. Most information derived from HCPs and experts was related to their understanding of cultural competence and its components. Both groups provided suggestions rather than specific items to capture the different dimensions of cultural competence. However, suggestions of HCPs and experts guided the selection of items from existing instruments.

Data were obtained from convenience samples of MS and PiA rather than from randomly selected practising HCPs. Although the study participants represent roughly 1% and 3% of the national MS and PiA population with characteristics fairly representative to the national sample [80, 81], the generalisability of the findings for other HCPs remains uncertain. It would also be important to conduct confirmatory factor analyses (CFA) to determine if the component structures from the former PCA holds true. Furthermore, group differences with regard to demographic characteristics, educational training, and scope of practice need to be considered. Both groups are taught communication skills, whereas cultural competence training is not a firm component of the curricula. Social and emotional skills development receives less attention in the medical curriculum than in psychotherapeutic training. On the other hand, practical skills are less standard in the psychotherapeutic context. Although the questionnaire was developed in a German healthcare setting, it is hypothesised that the CCCHP has the same informative value in other European countries. However, when used in other countries, the instrument should be checked on this, preferably not only by inspecting the psychometric properties but also on validity, e.g. by asking HCPs and experts’ opinion about the informative value.

An inherent limitation of the CCCHP and other self-report measures is the uncertainty to what extent these measures actually reflect professionals’ cultural competencies in practice, rather than professionals’ intentions and self-efficacy [39]. The additional use of independent instruments and qualitative methods (e.g. direct observation, objective structured clinical examination) that evaluate professionals’ cultural competence skills would be valuable.

Convergent and discriminant validity were not assessed in the course of this study. There was no validated instrument in German available that assessed a concept close to cultural competence in this particular context. Besides, the completion time for the 59-item online survey was 16 minutes and adding another instrument would have possibly reduced the sample size. However, further research is necessary to evaluate the convergent and discriminant validity of the CCCHP.

Cultural competence assessment relies to a great extent on self-report instruments. Self-report instruments are often susceptible to social desirability bias. Sodowsky [19] recommended administering cultural competence measures with a multicultural social desirability scale that has multicultural content and face validity. In the present study, the six SD items were adopted from a reliable and validated instrument (SDS-17) and modified to enhance their suitability for the target sample and context. In the PCA, only four of the six SD items formed the SD scale. These results indicate that not all items may be suitable for the sample. Hence, additional content and construct validity assessment of the modified SD scale is suggested. Moreover, the significant correlations between the priori defined SD scale and the CC-EE subscale, being an important component of cultural competence, warrant greater attention. The semantic content of these items might activate socially desirable responding. In a next step, CFA with the SD scale treated as external criterion is recommended to assess the extent that SD presents a threat to a valid interpretation of the cc subscale scores.

### Implications for practice

Given the current evidence of health and healthcare disparities within Europe [1], there is an increasing need for HCPs to provide culturally competent care to diverse patient populations. The CCCHP, after further validation, can assist HCPs to reflect on their behaviour, to reassess their knowledge and to gain a stronger awareness of their own strengths and weaknesses. Furthermore, periodic and systematic self-assessment can encourage professional development and continuous training, and thus contribute to improving the quality of patient care. The value of the CCCHP lies less in its ability to assess predictive performances of culturally competent behaviour, but more in its potential to identify starting points for further cultural competence development.

After responsiveness to change has been established, the CCCHP could serve as an efficient method to assess the effectiveness of training programs that are designed to enhance professionals’ cultural competence.

See **S1 File** for the original German version and **S2 File** for the English version of the CCCHP-27.

## Conclusions

The present study represents an effort to develop and evaluate an instrument to assess the cultural competence of HCPs. The results of this study suggest that the CCCHP is a multidimensional instrument composed of five dimensions of HCPs’ cultural competence. Further research is needed to confirm these findings in a random sample of practising HCPs.

The currently predominant conceptualisation of cultural competence refers to professionals’ attitudes, knowledge and skills in working with culturally diverse populations [32]. Moreover, the majority of cultural competence models have been developed in the United States. Qureshi and Collazos [21] emphasised that existing models need to be further defined, adapted, and researched for an effective application in the European context, where greater attention needs to be paid to immigration and cultural differences. As the CCCHP was developed in a German context, it can be the foundation to close this gap.

The development of the conceptual model for the CCCHP revealed Cross-Cultural Motivation/Curiosity and Cross-Cultural Emotions/Empathy as important components of cultural competence. With regard to the widespread tripartite model, an extended understanding of how professionals’ affective states may interact with those of the patients in the context of cross-cultural encounters seems helpful. The CCCHP can be used by HCPs themselves, as well as by managers and trainers.

As shown by the results attained in this study, the CCCHP can serve as a tool as well as a foundation for future research, in the area of cultural competence assessment for HCPs.

## Supporting Information

S1 Fig Overview of the study procedures for the development and psychometric evaluation of the CCCHP. (PDF)

S1 File Fragebogen zur Erhebung Interkultureller Kompetenz in der Gesundheitsversorgung (CCCHP-27) [Cross-Cultural Competence instrument for Healthcare Professionals (CCCHP -27)] *(original German version)*. (PDF)

S2 File Cross-Cultural Competence instrument for Healthcare Professionals (CCCHP-27) *(English version)*. (PDF)

S1 Methods Detailed description of methods. (DOCX)

## Abbreviations

CC-A: Cross-Cultural Attitudes
CC-EE: Cross-Cultural Emotions/Empathy
CC-KA: Cross-Cultural Knowledge/Awareness
CC-MC: Cross-Cultural Motivation/Curiosity
CC-S: Cross-Cultural Skills
CCCHP: Cross-cultural competence instrument for the healthcare profession
CCCI-R: Cross-Cultural Counselling Inventory-Revised
HCPs: Healthcare professionals
KMO: Kaiser-Meyer-Olkin
MAKSS/MAKSS-CE-R: Multicultural Awareness-Knowledge-and-Skills Survey
MCAS-B/MCKAS: Multicultural Counselling Knowledge and Awareness Scale
MCI: Multicultural Counselling Inventory
MS: Medical students
PCA: Principal component analysis
PiA: Psychologists in advanced psychotherapeutic training
QCA: Qualitative content analysis
SD: Social desirability
SDS-17: Social Desirability Scale-17

## References

[1] WHO Regional Office for Europe. How health systems can address health inequities linked to migration and ethnicity Copenhagen: WHO Regional Office for Europe; 2010.

[2] Yeo S. Language barriers and access to care. Annu Rev Nurs Res. 2004;22: 59–73. 15368768

[3] Canto JG, Allison JJ, Kiefe CI, Fincher C, Farmer R, Sekar P, et al Relation of race and sex to the use of reperfusion therapy in Medicare beneficiaries with acute myocardial infarction. N Engl J Med. 2000;342(15): 1094–1100. 10760310 10.1056/NEJM200004133421505

[4] Abreu JM. Conscious and Nonconscious African American Stereotypes: Impact on First Impression and Diagnostic Ratings by Therapists. J Consult Clin Psychol. 1999;67(3): 387–393. 10369059 10.1037//0022-006x.67.3.387

[5] Helms JE, Cook DA. Using race and culture in counseling and psychotherapy: Theory and process 1st ed. Needham Heights, MA: Allyn & Bacon; 1999.

[6] Bennegadi R. Cultural Competence and Training in Mental Health Practice in Europe: Strategies to Implement Competence and Empower Practitioners International Organization for Migration (IOM). Background Paper. Paris: Teaching and Research Department, Minkowska Centre 2009 Available at: http://www.migrant-health-europe.org/files/Mental%20Health%20Practice_Background%20Paper(1).pdf. Accessed December 16, 2014.

[7] Beach MC, Price EG, Gary TL, Robinson KA, Gozu A, Palacio A, et al Cultural Competence: A Systematic Review of Health Care Provider Educational Interventions. Med Care. 2005;43(4): 356–373. 15778639 10.1097/01.mlr.0000156861.58905.96 PMC3137284

[8] Gozu A, Beach MC, Price EG, Gary TL, Robinson K, Palacio A, et al Self-administered instruments to measure cultural competence of health professionals: A systematic review. Teach Learn Med. 2007;19(2): 180–190. 17564547 10.1080/10401330701333654

[9] Shen Z. Cultural Competence Models and Cultural Competence Assessment Instruments in Nursing: A Literature Review. J Transcult Nurs. 2014 5 9 doi: 10.1177/1043659614524790 10.1177/1043659614524790 24817206

[10] Deardorff DK. Identification and Assessment of Intercultural Competence as a Student Outcome of Internationalization. J Stud Int Educ. 2006;10(3): 241–266.

[11] Geiger HJ. Racial stereotyping and medicine: the need for cultural competence. CMAJ. 2001;164(12): 1699–1700. 11450212 PMC81156

[12] Campinha-Bacote J. The Process of Cultural Competence in the Delivery of Healthcare Services: A Model of Care. J Transcult Nurs. 2002;13(3): 181–184. 12113146 10.1177/10459602013003003

[13] Doorenbos AZ, Schim SM. Cultural competence in hospice. Am J Hosp Palliat Care. 2004;21(1): 28–32. 14748520 10.1177/104990910402100108

[14] Sue DW, Arredondo P, McDavis RJ. Multicultural Counseling Competencies and Standards: A Call to the Profession. J Couns Dev. 1992;70: 477–486.

[15] Lum D. A framework for cultural competence In Lum D. (Ed.) Culturally competent practice: A framework for understanding diverse groups and justice issues. 4th ed. Belmont, CA: Brooks/Cole; 2011 pp. 123–135.

[16] Black RM, Wells SA. Culture and Occupation: A Model of Empowerment in Occupational Therapy. Bethesda, MD: American Occupational Therapy Association; 2007.

[17] Stone JH. Culture and disability: Providing culturally competent services Thousand Oaks, CA: Sage Publications; 2005.

[18] Sue DW, Bernier JE, Durran A, Feinberg L, Pedersen P, Smith EJ, et al Position paper: Cross-cultural counseling competencies. Couns Psychol. 1982;10(2): 45–52.

[19] Sodowsky GR. The Multicultural Counseling Inventory: Psychometric properties and some uses in counseling training In: Sodowsky GR, Impara JC, editors. Multicultural assessment in counseling and clinical psychology. Lincoln, NE: Buros Institute of Mental Measurements; 1996 pp. 283–324.

[20] Hays DG. Assessing Multicultural Competence in Counselor Trainees: A Review of Instrumentation and Future Directions. J Couns Dev. 2008;86: 95–101.

[21] Qureshi A, Collazos F. Cultural competence in the mental health treatment of immigrant and ethnic minority clients. Diversity in Health & Social Care. 2005;2(4): 307–317.

[22] Jirwe M, Gerrish K, Emami A. The theoretical framework of cultural competence. J Multicult Nurs Health. 2006;12(3): 6–16.

[23] Jirwe M, Gerrish K, Keeney S, Emami A. Identifying the core components of cultural competence: findings from a Delphi study. J Clin Nurs. 2009;18(18): 2622–2634. doi: 10.1111/j.1365-2702.2008.02734.x 19538568 10.1111/j.1365-2702.2008.02734.x

[24] Boyle DP, Springer A. Toward a Cultural Competence Measure for Social Work with Specific Populations. J Ethn Cult Divers Soc Work. 2001;9(3): 53–71.

[25] Geron SM. Cultural Competency: How Is It Measured? Does It Make a Difference? Generations. 2002;26(3): 39–45.

[26] Sodowsky GR, Taffe RC, Gutkin TB, Wise SL. Development of the Multicultural Counseling Inventory: A self-report measure of multicultural competencies. J Couns Psychol. 1994;41(2): 137–148.

[27] LaFromboise TD, Coleman HL, Hernandez A. Development and factor structure of the Cross-Cultural Counseling Inventory-Revised. Prof Psychol Res Pr. 1991;22(5): 380–388.

[28] D'Andrea M, Daniels J, Heck R. Evaluating the Impact of Multicultural Counseling Training. J Couns Dev. 1991;70(1): 143–150.

[29] Kim BSK, Cartwright BY, Asay PA, D'Andrea MJ. A Revision of the Multicultural Awareness, Knowledge, and Skills Survey-Counselor Edition. Meas Eval Couns Dev 2003;36(3): 161–180.

[30] Ponterotto JG, Alexander CM. Assessing the multicultural competence of counselors and clinicians In: Suzuki LA, Meller PJ, Ponterotto JG, editors. Handbook of multicultural assessment: Clinical, psychological, and educational applications. San Francisco: Jossey-Bass; 1996 pp. 651–672.

[31] Ponterotto JG, Gretchen D, Utsey SO, Rieger BP, Austin R. A revision of the Multicultural Counseling Awareness Scale. J Multicult Couns Devel. 2002;30(3): 153–181.

[32] Constantine MG, Ladany N. New visions for defining and assessing multicultural counseling competence In: Ponterotto JG, Casas JM, Suzuki LA, Alexander CM, editors. Handbook of multicultural counseling. 2nd ed. Thousand Oaks, CA: Sage; 2001 pp. 482–498.

[33] Dunn TW, Smith TB, Montoya JA. Multicultural competency instrumentation: A review and analysis of reliability generalization. J Couns Dev. 2006;84(4): 471–482.

[34] Clark LA, Watson D. Constructing Validity: Basic Issues in Objective Scale Development. Psychol Assess. 1995;7(3): 309–319.

[35] Lynn MR. Determination and Quantification Of Content Validity. Nurs Res. 1986;35(6): 382–385. 3640358

[36] Worthington RL, Whittaker TA. Scale Development Research: A Content Analysis and Recommendations for Best Practices. Couns Psychol. 2006;34(6): 806–838.

[37] Liu M, Kunaiktikul W, Senaratana W, Tonmukayakul O, Eriksen L. Development of competency inventory for registered nurses in the People's Republic of China: scale development. Int J Nurs Stud. 2007;44(5): 805–813. 16519890 10.1016/j.ijnurstu.2006.01.010

[38] Mayring P. Qualitative Inhaltsanalyse: Grundlagen und Techniken [Qualitative content analysis: Fundamentals and techniques]. 10th ed. Weinheim: Beltz Verlag; 2008.

[39] Constantine MG, Ladany N. Self-Report Multicultural Counseling Competence Scales: Their Relation to Social Desirability Attitudes and Multicultural Case Conceptualization Ability. J Couns Psychol. 2000;47(2): 155–164.

[40] Stöber J. The Social Desirability Scale-17 (SDS-17) Convergent Validity, Discriminant Validity, and Relationship with Age. Eur J Psychol Assess. 2001;17(3): 222–232.

[41] Polit DF, Beck CT. Nursing Research: Principles and Methods 7th ed. Philadelphia, PA: Lippincott Williams & Wilkins; 2004.

[42] Berk RA. Importance of expert judgment in content-related validity evidence. West J Nurs Res. 1990;12(5): 659–671. 2238643 10.1177/019394599001200507

[43] Goodwin LD. Changing conceptions of measurement validity: An update on the new standards. J Nurs Educ. 2002;41(3): 100–106. 11939227 10.3928/0148-4834-20020301-05

[44] Grant JS, Davis LL. Selection and use of content experts for instrument development. Res Nurs Health. 1997;20(3): 269–274. 9179180 10.1002/(sici)1098-240x(199706)20:3<269::aid-nur9>3.0.co;2-g

[45] Razum O, Zeeb H, Meesmann U, Schenk L, Bredehorst M, Brzoska P, et al Migration und Gesundheit [Migration and Health] Schwerpunktbericht der Gesundheitsberichterstattung des Bundes. Berlin: Robert Koch-Institut, 2008 Available at: http://edoc.rki.de/documents/rki_fv/ren4T3cctjHcA/PDF/253bKE5YVJxo_28.pdf. Accessed February 9, 2011.

[46] Ferguson E, Cox T. Exploratory Factor Analysis: A Users’Guide. Int J Select Assess. 1993;1(2): 84–94.

[47] Lamping DL, Schroter S, Marquis P, Marrel A, Duprat-Lomon I, Sagnier P-P. The Community-Acquired Pneumonia Symptom Questionnaire: A New, Patient-Based Outcome Measure To Evaluate Symptoms in Patients With Community-Acquired Pneumonia. Chest. 2002;122(3): 920–929. 12226033 10.1378/chest.122.3.920

[48] Bartlett MS. A Note on the Multiplying Factors for Various x² Approximations. J R Stat Soc Series B Stat Methodol. 1954;16(2): 296–298.

[49] Kaiser HF. A second generation little jiffy. Psychometrika. 1970;35(4): 401–415.

[50] Kaiser HF. An index of factorial simplicity. Psychometrika. 1974;39(1): 31–36.

[51] Kline P. An Easy Guide to Factor Analysis. London: Routledge; 1994.

[52] Agius RM, Blenkin H, Deary IJ, Zealley HE, Wood RA. Survey of perceived stress and work demands of consultant doctors. Occup Environ Med. 1996;53(4): 217–224. 8664957 10.1136/oem.53.4.217 PMC1128453

[53] Tabachnick BG, Fidell LS. Using Multivariate Statistics. 5th ed. Boston, MA: Allyn and Bacon: Pearson Education Inc; 2007.

[54] Cattell RB. The scree test for the number of factors. Multivariate Behav Res. 1966;1: 245–276. 26828106 10.1207/s15327906mbr0102_10

[55] Fabrigar LR, Wegener DT, MacCallum RC, Strahan EJ. Evaluating the Use of Exploratory Factor Analysis in Psychological Research. Psychol Methods. 1999;4(3): 272–299.

[56] DeVellis RF. Scale Development: Theory and Applications. 2nd ed. Thousand Oaks, CA: Sage Publications; 2003.

[57] Schenk L, Ellert U, Neuhauser H. Children and adolescents in Germany with a migration background. Methodical aspects in the German Health Interview and Examination Survey for Children and Adolescents (KiGGS) [in German]. Bundesgesundheitsblatt, Gesundheitsforschung, Gesundheitsschutz. 2007;50(5–6): 590–599. 17514443 10.1007/s00103-007-0220-z

[58] Kline P. A Handbook of Test Construction: Introduction of Psychometric Design London: Methuen & Co.; 1986.

[59] Nunnally JC. Psychometric Theory. 2nd ed. New York, NY: McGraw-Hill; 1978.

[60] Cattell RB. The Scientific Use of Factor Analysis in the Behavioral and Life Sciences. New York: Plenum Press; 1978.

[61] Gorsuch RL. Factor Analysis. 2nd ed. Hillsdale, NJ: Lawrence Erlbaum Associates; 1983.

[62] Kline RB. Principles and practice of structural equation modeling 3rd ed. New York, NY: Guilford Press; 2011.

[63] Jones MC, Johnston DW. The derivation of a brief Student Nurse Stress Index. Work Stress. 1999;13(2): 162–181.

[64] Harkness JA. Comparative Survey Research: Goals and Challenges In: de Leeuw ED, Hox JJ, Dillman DA (Ed.). International Handbook of Survey Research Methodology. New York: Lawrence Erlbaum Associates; 2008 p. 56–77.

[65] Burns N, Grove SK. The Practice of Nursing Research: Conduct, Critique, and Utilization. 5th ed. St. Louis, Missouri: Elsevier Saunders; 2005.

[66] Schim SM, Doorenbos A, Benkert R, Miller J. Culturally Congruent Care: Putting the Puzzle Together. J Transcult Nurs. 2007;18(2): 103–110. 17416711 10.1177/1043659606298613

[67] Collins NM, Pieterse AL. Critical Incident Analysis Based Training: An Approach for Developing Active Racial/Cultural Awareness. J Couns Dev. 2007;85(1): 14–23.

[68] Byars-Winston AM, Fouad NA. Metacognition and Multicultural Competence: Expanding the Culturally Appropriate Career Counseling Model. Career Dev Q. 2006;54(3): 187–201.

[69] Priest J, McColl E, Thomas L, Bond S. Developing and refining a new measurement tool. Nurse Res. 1995;2(4): 69–81.

[70] Sue S. Cultural competency: From philosophy to research and practice. J Community Psychol. 2006;34(2): 237–245.

[71] Cronbach LJ, Meehl PE. Construct validity in psychological tests. Psychol Bull. 1955;52(4): 281–302. 13245896 10.1037/h0040957

[72] Campinha-Bacote J. The process of cultural competence in the delivery of healthcare services: A culturally competent model of care Cincinnati, OH: Transcultural C.A.R.E; 2003.

[73] Mason JL. Cultural Competence Self-Assessment Questionnaire: A Manual for Users. Portland: Portland State University, OR. Research and Training Center on Family Support and Children's Mental Health; 1995.

[74] Ponterotto JG, Burkard A, Rieger BP, Grieger I, D'Onofrio A, Dubuisson A, et al Development and Initial Validation of the Quick Discrimination Index (QDI). Educ Psychol Meas. 1995;55(6): 1016–1031.

[75] Sue S, Zane N. The role of culture and cultural techniques in psychotherapy: A critique and reformulation. Am Psychol. 1987;42(1): 37–45. 3565913 10.1037//0003-066x.42.1.37

[76] Gerrish K, Husband C, Mackenzie J. Nursing for a Multi-Ethnic Society. Buckingham, MK: Open University Press; 1996.

[77] Doorenbos AZ, Schim SM, Benkert R, Borse NN. Psychometric evaluation of the cultural competence assessment instrument among healthcare providers. Nurs Res. 2005;54(5): 324–331. 16224318 10.1097/00006199-200509000-00006

[78] Fuertes J, Bartolomeo M, Nichols CM. Future research directions in the study of counselor multicultural competency. J Multicult Couns Devel. 2001;29(1): 3–12.

[79] Pope-Davis DB, Liu WM, Toporek RL, Brittan-Powell CS. What's missing from multicultural competency research: Review, introspection, and recommendations. Cultur Divers Ethnic Minor Psychol. 2001;7(2): 121–138. 11381815 10.1037/1099-9809.7.2.121

[80] Statistisches Bundesamt. Bildung und Kultur Studierende an Hochschulen. Fachserie 11 Reihe 4.1, Wiesbaden: Statistisches Bundesamt (Destatis); 2010. 28. September 2010, korrigiert am 11. Oktober 2010.

[81] Strauß B, Barnow S, Brähler E, Fegert J, Fliegel S, Freyberger HJ, et al Forschungsgutachten zur Ausbildung von Psychologischen Psychotherapeuten und Kinder- und Jugendpsychotherapeuten Im Auftrag des Bundesministeriums für Gesundheit. Jena: Universitätsklinikum Jena; 4 2009.

