# Supplementary material for: Development and Psychometric Evaluation of an Instrument to Assess Cross-Cultural Competence of Healthcare Professionals (CCCHP)
Source: PLoS One. 2015 Dec 7;10(12):e0144049. doi: 10.1371/journal.pone.0144049 (PMC4671537; doi:10.1371/journal.pone.0144049)
Supplement: S1 Fig — (PDF) [file pone.0144049.s001.pdf]

Phase I: Instrument Development (CCCHP-EU)

Step 1

- International/National Expert Survey N=23
- Interviews Healthcare Professionals N=12

Qualitative Content Analysis

MODEL

Literature Review Conceptual Cultural Competence Models (14) → Model ← Literature Review existing Cultural Competence Instruments (20)

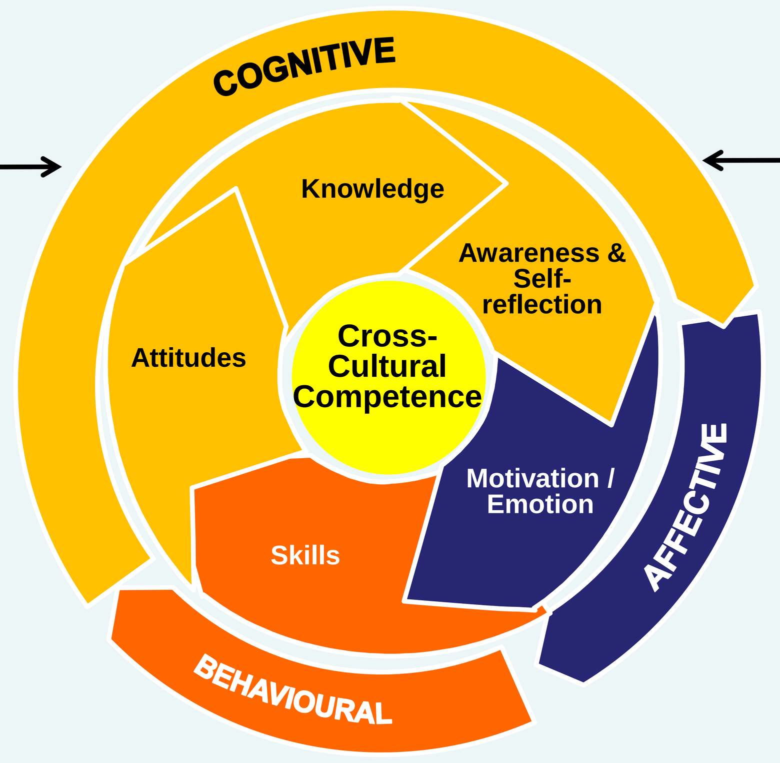

Step	Process	Details	Result
Step 2	Item Generation (384 items) and Reduction	4 item reduction rounds separately by 3 researchers	115 items & 6 SD items
Step 3	Initial Item Validation	13 researchers/students judged item relevance and clarity	85 items & 6 SD items
Step 4	Expert Validation	Face validity assessed by 5 experts	59 items & 6 SD items
Step 5	CCCHP-EU Pilot Version	**5 Dimensions:** Attitudes (11 items) Knowledge (13 items) Awareness/Self-reflection (10 items) Motivation/Emotion (12 items) Skills (13 items)	59 items & 6 SD items
Step 6	Usability testing CCCHP-EU Online Versions	Tested by 12 students	59 items & 6 SD items

Phase II: Psychometric Evaluation of the CCCHP-EU

Medical students (N=409) and Psychologists in advanced psychotherapeutic training (N=336)

Step	Process	Details	Result
Step 1	Pre-analysis checks & Item Analysis	>30% missing (2 items, 7 respondents)	63 items
Step 2	Principal Component Analysis (PCA)	<.39 component loading (24 items) >.40 cross-loading (3 items)	36 items
Step 3	Reliability (α)	4 items increased α estimate if deleted	32 items (4 SD)
Step 4	Additional Construct Validity Assessment	Known-groups technique 6 pre-defined groups	32 items (4 SD)

S1 Fig. Overview of the study procedures for the development and psychometric evaluation of the CCCHP-EU.
