# Supplementary material for: Development and Psychometric Evaluation of an Instrument to Assess Cross-Cultural Competence of Healthcare Professionals (CCCHP)
Source: PLoS One. 2015 Dec 7;10(12):e0144049. doi: 10.1371/journal.pone.0144049 (PMC4671537; doi:10.1371/journal.pone.0144049)
Supplement: S1 File — (PDF) [file pone.0144049.s002.pdf]

Fragebogen zur Erhebung Interkultureller Kompetenz in der Gesundheitsversorgung (CCCHP-27)

Universitätsklinikum Hamburg-Eppendorf (UKE)
Institut für Medizinische Psychologie
Arbeitsgruppe Psychosoziale Migrationsforschung
Martinistrasse 52
20246 Hamburg

Machen Sie bitte hinter jeder Aussage nur ein Kreuz in das Kästchen mit der für Sie am besten zutreffenden Antwort	Stimmt völlig	Stimmt eher	teils - teils	Stimmt eher nicht	Stimmt gar nicht	Keine Antwort möglich
1. Ich empfinde es als Bereicherung, in einem interkulturellen Team zu arbeiten.	❑5	❑4	❑3	❑2	❑1	❑0
2. Ich frage nach, was Patienten mit Migrationshintergrund an Unterstützung benötigen, um zum vereinbarten Behandlungsziel zu gelangen.	❑5	❑4	❑3	❑2	❑1	❑0
3. Ich finde es eine Zumutung, wenn Menschen, die schon vor langer Zeit nach Deutschland eingewandert sind, nicht richtig Deutsch sprechen können.	❑5	❑4	❑3	❑2	❑1	❑0
4. Innerhalb der Bevölkerungsgruppe mit Migrationshintergrund gibt es kaum Unterschiede in Bezug auf Gesundheitschancen und Krankheitsrisiken.	❑5	❑4	❑3	❑2	❑1	❑0
5. Ich kann etwas über andere kulturelle Orientierungen erfahren, wenn ich mit Patienten mit Migrationshintergrund kommuniziere.	❑5	❑4	❑3	❑2	❑1	❑0
6. Meine Wahrnehmung, Bewertung und mein Verhalten im Arbeitskontext bleiben durch meine kulturelle Prägung unbeeinflusst.	❑5	❑4	❑3	❑2	❑1	❑0
7. Kulturelle Vielfalt ist auch eine Bereicherung.	❑5	❑4	❑3	❑2	❑1	❑0
8. Ich spreche gerne mit Menschen mit Migrationshintergrund über deren Erfahrungen hier in Deutschland.	❑5	❑4	❑3	❑2	❑1	❑0
9. Es fällt mir häufig schwer, die Ausführungen meiner Patienten nachzuvollziehen, wenn ihr soziokultureller Kontext stark von meinem abweicht.	❑5	❑4	❑3	❑2	❑1	❑0
10. Ich mache keine Unterschiede zwischen den Patienten und behandle alle gleich, auch wenn es manchmal schwierig ist, sich zu verständigen.	❑5	❑4	❑3	❑2	❑1	❑0
11. Ich habe den Eindruck, dass Migranten oft Diskriminierungsabsichten unterstellen, wo es nur um die Durchsetzung allgemeiner Regeln gilt.	❑5	❑4	❑3	❑2	❑1	❑0
12. Der Migrationsprozess ist ein kritisches Lebensereignis und kann mit psychosozialem Stress und gesundheitlichen Belastungen einhergehen.	❑5	❑4	❑3	❑2	❑1	❑0

© Universitätsklinikum Hamburg-Eppendorf (UKE)

Universitätsklinikum Hamburg-Eppendorf

Machen Sie bitte hinter jeder Aussage nur ein Kreuz in das Kästchen mit der für Sie am besten zutreffenden Antwort	Stimmt völlig	Stimmt eher	teils - teils	Stimmt eher nicht	Stimmt gar nicht	Keine Antwort möglich
13. Langsames, für Laien verständliches Sprechen mit Menschen, die meine Anweisungen nicht gut verstehen können, fällt mir schwer.	❑5	❑4	❑3	❑2	❑1	❑0
14. Ich bleibe immer freundlich und zuvorkommend gegenüber Menschen mit anderen kulturellen Hintergründen, auch wenn ich selbst gestresst bin.	❑5	❑4	❑3	❑2	❑1	❑0
15. Die Begegnungen mit Menschen anderer kultureller Herkünfte helfen mir meine eigene Kultur genauer wahrzunehmen.	❑5	❑4	❑3	❑2	❑1	❑0
16. Die Krankheitskonzepte von Patienten mit Migrationshintergrund sind für den Behandlungserfolg nicht relevant.	❑5	❑4	❑3	❑2	❑1	❑0
17. In einem Gespräch lasse ich Menschen mit Migrationshintergrund stets ausreden und höre ihnen aufmerksam zu.	❑5	❑4	❑3	❑2	❑1	❑0
18. Im Streit mit Menschen mit anderen kulturellen Hintergründen bleibe ich stets sachlich und objektiv.	❑5	❑4	❑3	❑2	❑1	❑0
19. Ich würde gerne Fortbildungs-, Beratungs- und Schulungsangebote in Anspruch nehmen, um mein Verständnis mit Patienten mit Migrationshintergrund zu verbessern.	❑5	❑4	❑3	❑2	❑1	❑0
20. Ich finde es bereichernd, Freundschaften mit Menschen anderer kultureller Herkünfte zu haben.	❑5	❑4	❑3	❑2	❑1	❑0
21. Menschen, die nach Deutschland einwandern, sollten sich anpassen und nicht die Gesellschaft an die Zuwanderer.	❑5	❑4	❑3	❑2	❑1	❑0
22. Bei Patienten, die nicht so gut Deutsch verstehen, plane ich mehr Zeit ein, ihnen die Behandlungsmöglichkeiten verständlich zu machen.	❑5	❑4	❑3	❑2	❑1	❑0
23. Ich behandle lieber Patienten aus meinem eigenen kulturellen Kontext als Patienten, die mir fremd erscheinen.	❑5	❑4	❑3	❑2	❑1	❑0
24. Bei Patienten, die nicht so gut Deutsch verstehen, nehme ich mir mehr Zeit, um ihre Erwartungen und Befürchtungen zu besprechen.	❑5	❑4	❑3	❑2	❑1	❑0

© Universitätsklinikum Hamburg-Eppendorf (UKE)

Machen Sie bitte hinter jeder Aussage nur ein Kreuz in das Kästchen mit der für Sie am besten zutreffenden Antwort	Stimmt völlig	Stimmt eher	teils - teils	Stimmt eher nicht	Stimmt gar nicht	Keine Antwort möglich
25. Kulturspezifische Faktoren (z.B. Werte, Verhaltensnormen, Glaubenseinstellungen) von Menschen beeinflussen deren Krankheitsverständnis erheblich und sollten daher vom Behandelnden erfragt und beachtet werden.	❑5	❑4	❑3	❑2	❑1	❑0
26. Ich gehe auf Wertevorstellungen der Patienten bezogen auf Familie, Religion etc. ein, sofern mir diese relevant für die Versorgung erscheinen.	❑5	❑4	❑3	❑2	❑1	❑0
27. Im beruflichen Umgang mit Patienten mit Migrationshintergrund verspüre ich oft Verunsicherung, Ärger und Frustration.	❑5	❑4	❑3	❑2	❑1	❑0
28. Es ist mir ein Anliegen, Patienten ihren kulturellen Bedürfnissen und individuellen Werten entsprechend zu versorgen.	❑5	❑4	❑3	❑2	❑1	❑0
29. Auf die Sonderwünsche von Migranten wird in der Öffentlichkeit und in den Institutionen zu sehr Rücksicht genommen.	❑5	❑4	❑3	❑2	❑1	❑0
30. Ich zögere niemals, jemandem aus einem anderen kulturellen Kontext in einer Notlage beizustehen.	❑5	❑4	❑3	❑2	❑1	❑0
31. Ich werde ungeduldig, wenn ich mich mit Patienten mit Migrationshintergrund nicht verständlich machen kann.	❑5	❑4	❑3	❑2	❑1	❑0
32. Ich finde es spannend, Patienten mit Migrationshintergrund zu behandeln.	❑5	❑4	❑3	❑2	❑1	❑0

© Universitätsklinikum Hamburg-Eppendorf (UKE)

Skalen des „Fragebogens zur Erhebung Interkultureller Kompetenz in der Gesundheitsversorgung“ (CCCHP-27)

Der CCCHP-27 umfasst fünf Skalen Interkultureller Kompetenz. Die verschiedenen Skalenwerte werden als Mittelwert der entsprechenden Itemrohwerte bestimmt.

**Skalen Interkultureller Kompetenz**	**Item No.**
Motivation<br>(9 Items)	20, 7, 32, 1, 8, 15, 5, 19, 28
Einstellungen<br>(4 Items)	3*, 21*, 29*, 11*
Fähigkeiten<br>(5 Items)	24, 2, 22, 25, 26,
Emotionen/Empathiefähigkeit<br>(5 Items)	27*, 9*, 31*, 13*, 23*
Wissen/Bewusstsein<br>(4 Items)	16*, 4*, 6*, 12

* Die mit einem Stern gekennzeichneten Items müssen vor der Berechnung der Skalenwerte umkodiert werden (5=1, 4=2, 3=3, 2=4, 1=5).

Zusätzlich wurden 5 Items in den CCCHP-27 aufgenommen um den Einfluss von sozialer Erwünschtheit auf das Antwortverhalten zu erfassen.

Soziale Erwünschtheit<br>(5 Items)	30, 18, 14, 10, 17

© Universitätsklinikum Hamburg-Eppendorf (UKE)
