# Supplementary material for: Development and Psychometric Evaluation of an Instrument to Assess Cross-Cultural Competence of Healthcare Professionals (CCCHP)
Source: PLoS One. 2015 Dec 7;10(12):e0144049. doi: 10.1371/journal.pone.0144049 (PMC4671537; doi:10.1371/journal.pone.0144049)
Supplement: S2 File — (PDF) [file pone.0144049.s003.pdf]

Cross-**C**ultural **C**ompetence instrument for **H**ealthcare **P**rofessionals

(**CCCHP-27**)

University Medical Centre Hamburg-Eppendorf (UKE)
Department of Medical Psychology
Study Group on Psychosocial Migration Research
Martinistrasse 52
20246 Hamburg, Germany

After each statement, please only mark the box for the answer which is most applicable to you.	Completely agree	Mostly agree	Neither agree nor disagree	Mostly disagree	Completely disagree	No answer possible
1. I consider working in a cross-cultural team an enrichment.	❑5	❑4	❑3	❑2	❑1	❑0
2. In order to achieve the agreed treatment goal, I ask patients with a migration background what they need in terms of support.	❑5	❑4	❑3	❑2	❑1	❑0
3. I find it an imposition, when people who migrated to [country concerned] a long time ago, cannot speak [language concerned] properly.	❑5	❑4	❑3	❑2	❑1	❑0
4. Within the migrant population, there are hardly any differences in terms of health opportunities and disease risks.	❑5	❑4	❑3	❑2	❑1	❑0
5. By communicating with patients with a migration background I can learn about different cultural orientations.	❑5	❑4	❑3	❑2	❑1	❑0
6. My professional perception, assessment, and behaviour remain untouched by my cultural imprinting.	❑5	❑4	❑3	❑2	❑1	❑0
7. Cultural diversity is also an enrichment.	❑5	❑4	❑3	❑2	❑1	❑0
8. I enjoy talking to people who have migrated to [country concerned] about their experiences here.	❑5	❑4	❑3	❑2	❑1	❑0
9. I often find it difficult to relate to the elaborations of my patients, when their socio-cultural background is quite different from my own.	❑5	❑4	❑3	❑2	❑1	❑0
10. I do not differentiate between patients and treat all equally, even though it is sometimes difficult to communicate.	❑5	❑4	❑3	❑2	❑1	❑0
11. I have the impression that migrants often assume discrimination, when in fact general rules are simply being enforced.	❑5	❑4	❑3	❑2	❑1	❑0
12. The migration experience is a critical life event and can be accompanied by psychosocial stress and health burden.	❑5	❑4	❑3	❑2	❑1	❑0

© University Medical Centre Hamburg-Eppendorf (UKE)

After each statement, please only mark the box for the answer which is most applicable to you.	Completely agree	Mostly agree	Neither agree nor disagree	Mostly disagree	Completely disagree	No answer possible
13. I find it difficult to speak slowly in lay language with people who struggle to understand my instructions.	❑5	❑4	❑3	❑2	❑1	❑0
14. I always remain friendly and courteous with people from a different cultural background, even when I am stressed out.	❑5	❑4	❑3	❑2	❑1	❑0
15. The interaction with people from other cultural backgrounds helps me reflect upon my own cultural background.	❑5	❑4	❑3	❑2	❑1	❑0
16. The disease concepts of patients with a migration background are not relevant for treatment success.	❑5	❑4	❑3	❑2	❑1	❑0
17. In a conversation I always listen attentively and let individuals with a migration background finish their sentences.	❑5	❑4	❑3	❑2	❑1	❑0
18. During arguments with people from a different cultural background, I always remain factual and objective.	❑5	❑4	❑3	❑2	❑1	❑0
19. I would like to make use of training, advising and educational offers, in order to improve my understanding of patients with a migration background.	❑5	❑4	❑3	❑2	❑1	❑0
20. I consider it an enrichment to have friendships with people from different cultural backgrounds.	❑5	❑4	❑3	❑2	❑1	❑0
21. People who migrate to [country concerned] should adapt to society, not the other way around.	❑5	❑4	❑3	❑2	❑1	❑0
22. With patients who do not understand [language concerned] very well, I take more time to explain the treatment options to them.	❑5	❑4	❑3	❑2	❑1	❑0
23. I prefer treating patients from my own cultural background, than those who seem foreign to me.	❑5	❑4	❑3	❑2	❑1	❑0
24. With patients who do not understand [language concerned] very well, I take more time to discuss their expectations and fears.	❑5	❑4	❑3	❑2	❑1	❑0

© University Medical Centre Hamburg-Eppendorf (UKE)

After each statement, please only mark the box for the answer which is most applicable to you.	Completely agree	Mostly agree	Neither agree nor disagree	Mostly disagree	Completely disagree	No answer possible
25. Culturally specific factors of people (e.g. values, behaviour norms, beliefs) influence their understanding of disease significantly, and should therefore be assessed and taken into consideration by healthcare professionals.	❑5	❑4	❑3	❑2	❑1	❑0
26. I consider the values of patients in relation to family, religion, etc., if they seem relevant for the treatment.	❑5	❑4	❑3	❑2	❑1	❑0
27. In my professional interaction with patients with a migration background, I often feel unsure, angry and frustrated.	❑5	❑4	❑3	❑2	❑1	❑0
28. It is important for me to treat patients according to their cultural needs and individual values.	❑5	❑4	❑3	❑2	❑1	❑0
29. Institutions and the public pay too much attention to the special wishes of migrants.	❑5	❑4	❑3	❑2	❑1	❑0
30. I never hesitate to help someone with a different cultural background in case of emergency.	❑5	❑4	❑3	❑2	❑1	❑0
31. I get impatient when I cannot make myself understood with patients with a migration background.	❑5	❑4	❑3	❑2	❑1	❑0
32. I find it exciting to treat patients with a migration background.	❑5	❑4	❑3	❑2	❑1	❑0

Remark: the term “people with a migration background” can be replaced with “people who have migrated to [country concerned]”.

© University Medical Centre Hamburg-Eppendorf (UKE)

Scales of the „Cross-Cultural Competence instrument for Healthcare Professionals“ (CCCHP-27)

The CCCHP-27 includes five scales measuring cross-cultural competence. The scale values are determined as the means of the corresponding raw item scores.

**Scales of Cross-Cultural Competence**	**Item No.**
Motivation/Curiosity<br>(9 Items)	20, 7, 32, 1, 8, 15, 5, 19, 28
Attitudes<br>(4 Items)	3*, 21*, 29*, 11*
Skills<br>(5 Items)	24, 2, 22, 25, 26,
Emotions/Empathy<br>(5 Items)	27*, 9*, 31*, 13*, 23*
Knowledge/Awareness<br>(4 Items)	16*, 4*, 6*, 12

* Items marked with an asterisk must be recoded before calculating the scale scores (5=1, 4=2, 3=3, 2=4, 1=5).

In addition, the CCCHP-27 also comprises five items for detecting the impact of social desirability on the responses.

Social desirability<br>(5 Items)	30, 18, 14, 10, 17

© University Medical Centre Hamburg-Eppendorf (UKE)
