# Supplementary material for: Development and Psychometric Evaluation of an Instrument to Assess Cross-Cultural Competence of Healthcare Professionals (CCCHP)
Source: PLoS One. 2015 Dec 7;10(12):e0144049. doi: 10.1371/journal.pone.0144049 (PMC4671537; doi:10.1371/journal.pone.0144049)
Supplement: S1 Methods — (DOCX) [file pone.0144049.s004.docx]

**S1 Methods. Detailed description of methods.**

The development and psychometric evaluation of the cross-cultural competence instrument for the healthcare profession (CCCHP) involved a systematic process (34-37) and proceeded in two phases with ten steps: 1) instrument development phase including six steps, and 2) psychometric evaluation phase including four steps (**S1 Fig.**). Methods and results for each phase will be presented sequentially.

**Phase 1: Instrument development**

In line with Lynn (35) and Liu et al. (37), instrument content was established by a review of the cultural competence literature and existing instruments, an expert survey and interviews with HCPs. Subsequently, items were generated to constitute a preliminary instrument.

Step 1 began with a narrative literature review conducted in December 2011 1) to identify conceptual models and existing instruments of cultural competence within a healthcare setting, 2) to identify the construct and content domains distinguished, and 3) to locate items in existing instruments. Computerized searches of 12 bibliographic databases (CINAHL / PsycINFO / PsycArticles / SocINDEX via Ebsco, EMBASE / ERIC / Medline via Ovid, PubMed, SAGE Journals Online, Psychology and Behavioural Sciences Collection, SpringerLink, Web of Sciences), Google Scholar (including technical reports) and hand searches yielded more than 900 studies. using combinations of the following keyword roots (English): c*ultur, cross-cultur, intercultur, multicultur, competenc, sensitiv, capab, model, theory, framework, assess, instrument, scale, test, evaluat, tool, health, nursing, counsel*. Additional studies were identified by expanding database searches to include cultural competence instrument names and by scanning the reference lists of key review articles. A full and comprehensive search of the reference lists was not carried out. Articles were excluded if they were not written in English or German, published before 1980, did not include some form of cultural competence theory, conceptual model or quantitative cultural competence assessment, did not target healthcare providers or related professions, did not have a full article available for review, were duplicates of another article, or did not apply to any of the study questions.

More than 120 peer-reviewed articles on cultural competence models were identified. Eventually, 14 articles representing unique cultural competence models were retrieved. The most common components of the identified models were cognitive and behavioural components, with few addressing contextual elements. Cultural competence models were later used to guide the development of the CCCHP model.

The database and hand searches yielded 135 citations on cultural competence assessment instruments. In addition to reviewing articles on cultural competence instruments, an attempt was made to obtain and review all original instruments. For each instrument located, information on the instrument’s name, target population, number of items, dimensions and response scale were retrieved. Twenty-nine instruments were fully available, whereof the vast majority were self-report forms (93%), originated from the United States (86%) and were developed for the field of nursing (21%), counselling (28%) or healthcare (28%). The instruments ranged from 19 to 91 items, and their number of dimensions ranged from one to seven. The majority focused on cultural skills, awareness, and knowledge, whereas some also measured one or more of the following: multicultural counselling relationship, cultural desire, cultural encounters, flexibility/openness, emotional resilience, and cultural sensitivity. Two researchers (G.L., M.M.) assessed the instruments separately as to 1) their purpose (designed to evaluate cultural competence of individual HCPs), 2) relevance to healthcare practice in Germany, 3) psychometric properties, 4) conceptualisation of cultural competence, and 5) items used to represent the domains. The researchers judged the appropriateness of each instrument on a 3-point rating scale (not relevant to relevant). Disagreements on instrument appraisal were resolved by consensus. Six instruments were rated relevant, 14 were rated somewhat relevant and nine instruments were excluded as they were found to be irrelevant.

Next, an expert survey and interviews with different HCPs were performed. This approach ensured that a broad range of perspectives were included to inform the development of a conceptual model for the CCCHP. Experts and HCPs were asked to 1) define, from their perspective, cultural competence of HCPs, 2) identify the relevant components of cultural competence and 3) suggest items to capture the relevant components.

In total, 23 experts (response rate: 18%) participated in an online survey. The vast majority of respondents were from Germany (n=21), one third had a migration background, six directly provided healthcare services to patients, three were active cross-cultural trainers and most respondents (n=16) were engaged in research work.

To explore the perspectives of practising HCPs, semi-structured interviews (G.L.) were conducted with physicians, medical specialists, registered nurses, midwives and psychotherapists from healthcare settings in Hamburg, Germany. These HCPs were purposefully selected as they represent the target group for the CCCHP. Professionals were required to be working with culturally diverse patients. Twelve HCPs (response rate: 60%) gave their written informed consent to be interviewed and digitally recorded. Interviews lasted between 30 and 60 minutes, were transcribed verbatim and rendered anonymous. Of the 12 respondents, aged 31 to 60 (mean age: 44; SD=7.58), about half had a migration background and half participated in cultural competence training.

Data analyses were carried out separately for the expert survey and HCPs interviews by G.L. using qualitative content analysis (38). Within the iterative process, the categories were revised, reduced to main categories, and then verified with respect to their reliability (38). Both the material and the category system were checked by M.M. and discussed by a team of researchers (n=3). After extensive comparison of the themes in both the HCP and expert group, the researchers determined that the data exhibited common themes. Consequently, themes were combined to achieve a collective perspective. The results of this QCA were the basis for the development of the CCCHP cultural competence model.

Sixteen main categories of cultural competence derived from the QCA were grouped into dimensions by the research team. The inclusion of categories was determined by the absolute frequency of a topic and the consensus of experts and HCPs. These categories were structured into five dimensions: attitudes, knowledge, awareness/self-reflection, motivation/emotion, and skills. These dimensions were further classified into the three domains: cognitive, behavioural, and affective (**S1 Fig. Step 1**).

In Step 2, items were generated based on suggestions of the experts and HCPs as well as the 20 cultural competence instruments rated as relevant or somewhat relevant. For reasons of conceptual equivalence, only those items were selected which captured or could be rephrased and translated to capture one of the components of the CCCHP’s conceptual model. Statements provided by experts and HCPs guided the selection of items from existing instruments. An initial item pool of 384 items was generated, oriented on the instruments (81%), items from experts (6%) and from HCPs (13%). Only individual items were included from existing instruments; scales or subscale as a whole were not incorporated. The item pool was systematically reduced in four assessment rounds by three researchers (GL, MM, DD). Discrepancies in ratings were resolved through discussion. In the first round, 124 items (32%) were eliminated as they were found to not be representative of the content domain, not relevant to healthcare practice, or redundant. Next, the 260 items were reviewed concerning their appropriateness and clarity. Items were rated on a 3-point scale (not relevant/clear to relevant/clear). Items were eliminated if two or more researchers rated the items as not relevant, if items were clear duplicates or poorly worded. Items rated not relevant by only one researcher (14%) were discussed and by consensus either rejected or retained. After the second round, 126 items (49%) were eliminated. The remaining 134 items which were rated as “relevant with alteration” (items: 115/86%), were translated and cultural adaptations made, to suit the German healthcare system usage and response options. The researchers who were also fluent in English performed the forward translations. Challenging phases or uncertainties were resolved through discussions between the researchers. The first draft of the instrument included translated and/or adapted items combined with their original counterpart and response format. The researchers judged the draft instrument separately regarding item relevance, comprehensibility and items potential susceptibility to social desirability. Nineteen (23%) items were deleted. In addition, revisions to the wording were made to facilitate comprehensibility and cultural relevance of the translation.

It has been stressed that self-report instruments of cultural competence may be susceptible to social desirability (39). To identify individual cultural competence (cc) items that are prone to elicit socially desirable responding, six items (SD markers) of the Social-Desirability Scale-17 (SDS-17) (40) were found to be appropriate in terms of content (face validity) and were modified (i.e. culturally adjusted) for inclusion in the CCCHP.

A 5-point Likert Scale (fully labelled from strongly agree to strongly disagree) response format with a ‘no answer possible option’ was selected for the 115 cc items and 6 SD items to measure the level of agreement towards each item. The response format was deemed most appropriate as it allowed a neutral midpoint for respondents who may truly be indifferent with their degree of agreement regarding an item.

In Step 3, the draft 115-item instrument and the 6 SD items were administered to a convenience sample (41) of researchers and psychology students (n=13). Respondents judged how representative the individual items were of the construct content domain (42); evaluated item clarity; checked for redundancies and suggested revisions for item/instrument construction. Resulting from this review, 22 (19%) cc items were changed for greater clarity and 30 (26%) cc items were deleted or merged. This work yielded an 85-item instrument (plus 6 SD items) that was provided to experts for validation.

Step 4 served to establish face validity of the CCCHP’s content. An expert panel was used to evaluate whether the items measure what they were intended to measure (43). Quantitatively, experts were asked to judge 1) the relevance of each item to measure the respective dimension (42), and 2) if the item was correctly classified to capture the respective dimension. Qualitatively, comments on the clarity and meaning of item construction and wording were also elicited, including suggestions for modifications and refinement (42, 44). Five (response rate: 33%) experts participated in the validation process. Content validity testing resulted in the elimination of 23 (27%) cc items rated as ‘not relevant’ and in the rejection of eight (9%) cc items identified as redundant. The written feedback of experts resulted in the modification of 22 (26%) cc items, as they were rated ‘relevant with minor revisions’ and in the generation of five new cc items to complement the subscales.

Step 5 yielded the 59-item self-report CCCHP which is distributed across five dimensions: attitudes (11 items), knowledge (13 items), awareness and self-reflection (10 items), motivation (curiosity) and emotion (fear) (12 items), and skills (13 items). Five of these items originated from existing instruments. Six SD items were embedded to identify individual cc items that contained socially desirable content. Each item was measured with a 5-point Likert-type response scale from 1=strongly agree to 5=strongly disagree and a ‘no answer possible’ option. Twenty cc items were presented in reverse to prevent tendencies towards the response set and were randomly arranged throughout the scale. Sum scores are calculated for each subscale. The issue of weighting items is not addressed yet.

In Step 6, two online surveys of the CCCHP (only differing in background information) were established for the groups of respondents in the psychometric survey. The CCCHP-59 as well as the 6 SD items were presented in a forced-choice response format. To examine usability, the online surveys were pre-tested by 12 psychology students.

**Phase 2: Psychometric evaluation of the CCCHP**

**Study participants**

After minor revisions, the 59-item CCCHP was tested for its psychometric properties with two prospective HCP groups: medical students (MS; ≥5^th^ semester and thus in the clinical part of their studies and practical year) and psychologists in advanced psychotherapeutic training (PiA). Both groups were selected as they constitute the later target group for the CCCHP and have from this stage of their professional development patient contact and clinical experience. In addition to the CCCHP, socio-demographic and occupational/educational information were collected anonymously for the psychometric survey. The information collected included respondent’s age and gender, psychotherapeutic/medical training, migration background (45), frequency of patient contact, cross-cultural encounters and if respondents had prior diversity training. To assess baseline demographic differences between the two groups the independent t-test and chi-square analyses were computed.

An email invitation explaining the purpose of the study was sent to student representatives and dean’s offices of Medical Universities throughout Germany (n=34). Additionally, invitations were posted in online forums and medical students’ groups. To reach PiAs, requests were sent to PiA-network-providers, to PiA-forums and to all 180 PiA training institutions in Germany. As an incentive, participants were offered the opportunity to register for a lottery to win one of ten €20 gift certificates.

All participants received written information about the purpose and methods of the study. Participants were informed that their participation was voluntary and that they could refuse to participate or withdraw from the study at any time. Data were collected and handled anonymously. Completion of the online survey constituted informed consent.

**Data analysis**

Analyses focused on testing1) the suitability of the data set, 2) the instrument’s dimensionality (PCA; principal component analysis), 3) reliability, and 4) discriminating power (known-groups technique) (41) undertaken with the Statistical Package for Social Sciences version 18.0 (SPSS® Inc., Chicago, IL, USA).

In Step 1, pre-analysis checks were executed to ensure the suitability of the data set for factor analysis (46). The checks included determining the stability of the emerging factor structure, adequacy of sample size, item scaling, skewness and kurtosis of item distribution and the appropriateness of the correlation matrix. Descriptive statistics were used for background information.

To evaluate the instruments practicability and acceptance, response rates, time to complete and feedback from respondents were analysed. Acceptance was judged by calculating the completion rate for each item and the item-response distribution. In the statistical analyses, the ‘no answer possible’ option (forced-choice responses) was coded as missing. Missing values of ≥30% in any item or respondent were considered to be inadequate for inclusion and were thus eliminated from further analysis. Concerning each item’s distribution, the maximum acceptable proportion of floor and ceiling effects among items as well as subscales was <80% (47).

In Step 2, PCA were performed on the CCCHP-59 (plus 6 SD items) with orthogonal rotation (varimax with Kaiser normalisation) to determine the dimensionality of the instrument. Prior to PCA, the suitability of the data for factor analysis was assessed by the application of Bartlett’s test of sphericity (48) and the Kaiser-Meyer-Olkin (KMO) measure of sampling adequacy (49). A KMO value of 0.60 (50) and a significant Bartlett’s test (p <.05) were deemed to be minimum requirements to proceed with factor analysis.

Criteria for extraction of CCCHP components as proposed by Kline (51) and others (52, 53) included: 1) eigenvalues >1, 2) Cattell’s (54) scree test, 3) interpretability of the solution, using a component loading cut-off of .39 and no cross-loadings greater than or equal to .40, and 4) percentages of explained variance (minimum of 5% reported variance) per component. Moreover, methodologists have recommended that at least three to five items represent each component (55). In this study, the minimum amount of items loading on each component was set to four. Items were excluded if they met one of the following criteria: 1) weak loadings (failing to load above .39 on any component), 2) general loadings of .40 on more than one component, 3) ≥30% of responses were missing or 4) ≥80% of item responses were the same (floor/ceiling effect). Item reduction and subscale modification were performed based on the PCA.

In Step 3, internal consistency reliability was assessed for the CCCHP using Cronbach’s α to address the homogeneity of items in a scale. The criteria for Cronbachs α as described by DeVellis (56) were applied for the interpretation of results (α of >.70). The SD items were included in the PCA 1) to evaluate the reliability and factorial validity of the reduced and modified SD scale and 2) to identify cc items with qualities of SD items.

In Step 4, in addition to PCA, construct validity of the CCCHP was assessed by using the known-groups technique (41). Three subgroups were predefined based on theoretically expected differences in cultural competence. On the assumption that having a migration background, frequent cross-cultural encounters and participating in cross-cultural competence training would lead to higher scores on the CCCHP subscales, mean scores were calculated. Higher mean scores indicate a higher motivation, more positive attitudes and emotions, greater frequency of using culturally competent skills, and higher levels of knowledge. Higher mean scores on the SD subscale indicate a higher tendency to provide socially desirable responses. The independent sample t-test was used to analyse differences between group means on each CCCHP subscale and the SD subscale (37).

**Ethics Statement**

This study is part of the international research project on ‘Mental Health and Migration’ (www.segemi.de), approved by the Ethics Committee of the Hamburg Chamber of Psychotherapists, Germany. All participants received written information regarding the study according to the principles outlined in the Declaration of Helsinki. In the online psychometric survey, the written information contained a passage informing the participants that they declare their consent by completing the CCCHP-59.
